# Improving the Accuracy of Permeability Data to Gain Predictive Power: Assessing Sources of Variability in Assays Using Cell Monolayers

**DOI:** 10.3390/membranes14070157

**Published:** 2024-07-14

**Authors:** Cristiana L. Pires, Maria João Moreno

**Affiliations:** 1 Coimbra Chemistry Center—Institute of Molecular Sciences (CQC-IMS), University of Coimbra, 3004-535 Coimbra, Portugal; 2 Chemistry Department, Faculty of Science and Technology, University of Coimbra, 3004-535 Coimbra, Portugal

**Keywords:** permeability assays, drug bioavailability, Caco-2 monolayers, experimental variability, QSPRs, large datasets

## Abstract

The ability to predict the rate of permeation of new compounds across biological membranes is of high importance for their success as drugs, as it determines their efficacy, pharmacokinetics, and safety profile. In vitro permeability assays using Caco-2 monolayers are commonly employed to assess permeability across the intestinal epithelium, with an extensive number of apparent permeability coefficient (*P*_app_) values available in the literature and a significant fraction collected in databases. The compilation of these *P*_app_ values for large datasets allows for the application of artificial intelligence tools for establishing quantitative structure–permeability relationships (QSPRs) to predict the permeability of new compounds from their structural properties. One of the main challenges that hinders the development of accurate predictions is the existence of multiple *P*_app_ values for the same compound, mostly caused by differences in the experimental protocols employed. This review addresses the magnitude of the variability within and between laboratories to interpret its impact on QSPR modelling, systematically and quantitatively assessing the most common sources of variability. This review emphasizes the importance of compiling consistent *P*_app_ data and suggests strategies that may be used to obtain such data, contributing to the establishment of robust QSPRs with enhanced predictive power.

## 1. Introduction

**Figure 1:**
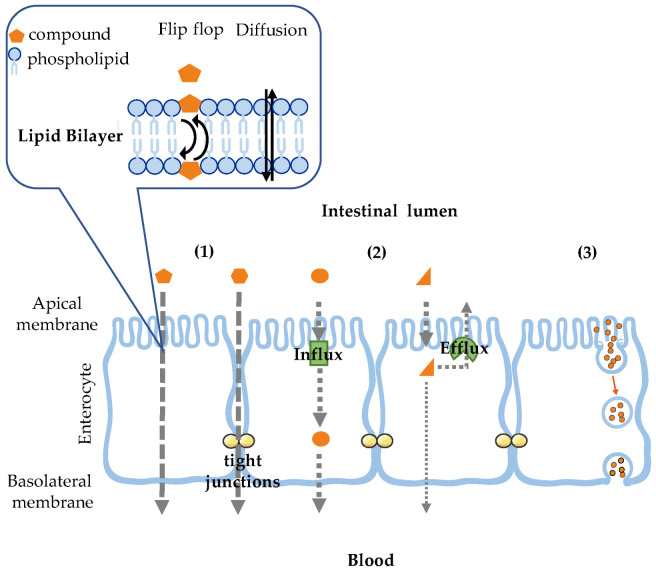
Mechanisms of drug permeation through a cell monolayer, exemplified for the case of the intestinal epithelium. (1) Passive diffusion occurs through the cell’s membrane (inset) or via the paracellular pathway between adjacent cells; (2) Carrier-mediated transport occurs for molecules that are recognized by transport proteins at the membrane. Depending on the direction of the transport, it may enhance (identified as Influx) or reduce (identified as Efflux) the cellular uptake of molecules; (3) Transcytosis occurs when large volumes of the aqueous medium outside the cell monolayer are moved through the cell inside of vesicles. This figure was produced in Microsoft PowerPoint using Servier Medical Art templates licensed under a Creative Commons Attribution 3.0 Unported License (https://smart.servier.com, accessed on 25 March 2024).

The assessment of pharmacokinetic (ADME—absorption, distribution, metabolism, and excretion) and toxicity profiles of new molecules is a necessary part of the optimization processes in order to arrive at a safe and efficacious drug [1]. When determining the ADME profile, the permeability across membranes is a fundamental property to be evaluated. Along with being a pre-requisite for good oral absorption, the permeability is crucial for the distribution of the drug to target organs and cells, as well as for its excretion, which involves crossing tissues and cell layers at the liver and kidney [2]. The most significant biological membranes in drug ADME are the epithelial and endothelial cell monolayers lining the intestinal tract and blood capillaries, respectively. Drugs permeate across a cell monolayer by several mechanisms, as shown in Figure 1. These pathways are distinct, and the molecular properties of the drug that influence its transport by these routes are also different [2,3,4]. Passive diffusion (1) defines the movement of molecules in response to their concentration gradient between the water medium on both sides of the membrane. This transport may occur by two pathways—though the cell (transcellular transport) or though the spaces between adjacent cells (paracellular transport). Small and very hydrophilic molecules may permeate paracellularly, diffusing through the tight junctions or gaps between the cells. This pathway shows some charge selectivity due to the electrostatic properties of the claudins present in the tight junctions [5,6,7,8,9,10]. In the transcellular transport, the molecule partitions into the outer lipid leaflet of the bilayer, and depending on the molecule structural features, it diffuses or translocates (flip-flops) from the outer to the inner lipid leaflet [11,12,13,14,15,16]. From there, the molecule equilibrates with the aqueous medium inside the cell, thus permeating the cell cytoplasmic membrane [17]. The molecular features that have traditionally been associated with the rate of passive transcellular permeation are lipophilicity and size, in tandem with the partition–diffusion model enunciated more than 100 years ago by Meyer and Overton [16] and incorporated in Lipinski’s rule of five [18]. Several exceptions, however, have been identified over time, and it is now fairly well established that permeation through this pathway is also influenced by solute conformation, the orientation and location in the membrane, as well as by its membrane-perturbing effects [12,13,19,20,21,22,23,24,25,26,27,28]. Carrier-mediated transport (2) defines the translocation of molecules across membranes using specialized transporters embedded in the lipid bilayer for which the molecules are ligands. The carrier proteins can be localized in the apical and basolateral sides of the cell monolayer, leading to transport through the cell monolayer, or be located only in one of the sides, leading to transport into the cell (influx) or out of the cell (efflux). A high solute specificity is observed for this permeation pathway. For very hydrophilic solutes, specificity is mostly dependent on the interactions established with the transporter protein, while interactions with the lipid membrane are also important for ligands with moderate-to-low hydrophilicity [29,30,31,32,33]. Transcytosis (3) defines the cellular internalization and trafficking process of a molecule associated with membrane vesicles. The overall process involves the formation of endocytic vesicles on one side of the cell monolayer, vesicle transport through the cell, and their exocytosis on the opposite side. This pathway is most relevant for the permeation of large entities (macromolecules, nanoparticles, and viruses) and usually involves the recognition of the transported entities by receptors that may be protein- or lipid-dependent [34,35,36,37,38,39,40].

## 2. The Need to Quantitatively Predict the Permeability from a Compound’s Chemical Structure

In vitro tests of medium-to-high-throughput are often used for screening permeability properties at early stages in drug discovery processes [41,42]. However, the market’s constant demand for more effective drugs has led to an exponential increase in the number of new chemical entities (NCEs) that need to be evaluated at this stage. This scaling up has increased the number of experimental permeability in vitro tests that need to be performed, raising costs and slowing down the discovery process [43,44]. This has raised the need for tools capable of virtual screening for a compound’s permeability before its synthesis. At this stage, computational tools (in silico) are the only option. To be able to generate such predictive models, it is necessary to understand the rules that relate permeation through the biological barriers and the compounds’ physicochemical properties, which in turn depend on their chemical structure.

Lipinski and coworkers were pioneers in the effort of identifying the relevant molecular structural features, also known as molecular descriptors, that correlate with oral bioavailability [45]. In doing so, the authors created a model using simple rules based on molecular descriptors that may easily be calculated, the well-known Lipinski’s rule of 5. According to those rules, poor absorption and membrane permeability are more likely to occur when molecules fulfil two or more of the following criteria: M_W_ > 500 Da; water–octanol partition coefficient (LogP) > 5; number of hydrogen bond donors (sum of O-Hs and N-Hs) > 5; and number of hydrogen bond acceptors (sum of N and O atoms) > 10. Those simple rules have been very useful in the screening of libraries of compounds. It should, however, be noted that they are a qualitative predictor of whether oral absorption is good or bad, incorporating the need for a moderate solubility in aqueous media and a moderate affinity for the membranes. Furthermore, these rules have been proposed as guidelines only, with many exceptions being identified where good absorption after oral administration is observed in spite of the unfavorable compound structural features (notably, the case of several antibiotics) [21,46,47,48,49,50,51,52,53].

Alongside the continuous increase in computing power and the growth of experimental data for many compounds, in silico modeling has evolved into more robust approaches aimed at quantitatively predicting drug bioavailability [54]. This would allow for going from a qualitative evaluation of whether the compound is likely to be efficiently absorbed towards a quantitative prediction of its availability, thus providing a better understanding and allowing for the fine tuning of this important property. These efforts have been focused on the prediction of solubility in the aqueous media, as well as on permeability through the intestinal epithelium membranes, which represents the major barrier for drug absorption. Mathematical rules denominated quantitative structure–property relationships (QSPRs) that attempted to relate membrane permeability to the compound’s molecular features have been proposed [55,56,57,58,59,60,61]. Kinetic modelling has also been used to accommodate distinct steps in the overall process of membrane permeation and drug bioavailability [11,12,17,59,62,63,64,65,66,67,68,69,70]. The attractiveness of this strategy is undisputable, as the property’s values of new compounds could be predicted even before their synthesis. A major additional advantage of establishing quantitative relationships is that it allows for extrapolations towards unexplored regions in drugs’ chemical space, providing insights for the design of new generations of bioactive molecules.

The expectations of in silico modelling applied to early discovery are far-reaching, facilitating the drug discovery process in many ways. First, in the design stage of NCEs and compound libraries. Having the ability to predict the effect of a given structural modification on permeability, these in silico models can be used to guide the rational design of molecules with improved properties from the beginning of the process. Also, smaller and more focused combinatorial libraries can be created to contain subsets of molecules with desirable properties and structural diversity. Second, in silico modeling is also useful in the optimization of the screening and testing of NCEs. By filtering the molecules, the models can lead to the rational selection of the most promising compounds for synthesis and enable the prioritization of the molecules that are tested both in vitro and in vivo [44].

## 3. Importance of the Consistency and Accuracy of Permeability Data to the Performance of QSPRs Models

The collection of accurate and consistent permeability data is essential to obtaining reliable permeability datasets for use in QSPRs and in silico modelling in general. Such data should have few experimental errors and, ideally, should be measured under identical experimental conditions, especially when compiled from a variety of sources including in vivo and in vitro assays [71].

### 3.1. QSPRs Derived from In Vivo Datasets

In vivo methods provide the most reliable measurements for assessing a compound’s bioavailability. However, in vivo data-based relationships are difficult to establish owing to the many steps and processes involved and the complexity of the biological systems. As discussed in Section 1, permeability through the intestinal barrier may occur via several pathways, including passive, facilitated, and active mechanisms, each depending differently on compounds’ molecular features. Additionally, on the way from the intestinal lumen to the blood stream, the compound may be chemically modified by enzymes present in the intestinal tract and gut wall and suffers the effects from the first passage through the liver [72,73]. Bioavailability is therefore not simply and directly related with permeability. Although bioavailability may be the end property to optimize, its complex relation with the compound properties will increase the difficulty in the establishment of QSPRs. Higher levels of success are anticipated if permeability and metabolism are considered and optimized independently. The collection of in vivo data is also experimentally demanding and expensive, limiting their use for only a small number of pre-selected, more promising compounds. The dataset is therefore usually biased towards compounds that are expected to show a good bioavailability [74,75,76]. This limits the chemical space analyzed and generates difficulties in the training and validation of reliable models [77]. Nevertheless, a few QSPRs models for oral bioavailability can be found in the literature.

Turner et al. [78] used oral bioavailability data for 159 structurally diverse drugs to develop a QSPR. The model obtained was applied to predict the bioavailability of a test set (*n* = 10), leading to a correlation value of 0.72. The relevant molecular descriptors identified include LogP and the polar surface area, both with a negative contribution, and Hansen’s hydrogen-bonding solubility parameter—in this case, with a positive contribution to bioavailability. A very strong negative correlation was also observed with the electron delocalization (HOMO energy), reflecting the compound reactivity and susceptibility to enzymatic metabolism.

In another work, Tian et al. [79] used a larger dataset including 1014 drugs to build a model for bioavailability that was evaluated on a separate test set of 80 compounds, leading to a correlation value of 0.71 when considering 110 descriptors. Poor correlations were observed between the intestinal absorption and oral bioavailability of the compounds in the dataset. Not surprising, the inability of simple rules to characterize the effect of intestinal absorption and metabolic processes on bioavailability leads to models with very poor performance when considering a small number of molecular descriptors (|r| < 0.337, 0.351, and 0.38, for 1, 2, and 7 descriptors, respectively). The single best descriptor was the number of flexible rotatable bonds, followed by the molecular weight (r = −0.324), both contributing negatively to bioavailability. Descriptors related with hydrophobicity showed a positive contribution but with very low correlations (r = 0.025 for LogP and r = 0.117 for LogD_5.5_), while a moderately better correlation was observed for descriptors directly related with hydrophilicity (r = −0.287 for the polar surface area and −0.261 for the number of H bonds as donors).

As discussed by Cabrera-Pérez et al. [74], due to the enormous complexity of the in vivo systems, the establishment of simple QSPRs from in vivo data is still a challenge. Also, the large diversity of molecular descriptors considered and their inter-dependence complicate the interpretation of the QSPR models in terms of specific molecular properties. This may be clearly seen with respect to the descriptor LogP, usually considered as a good predictor of lipophilicity and thus expected to contribute positively to intestinal absorption [45], while it was found to be not statistically relevant for bioavailability in the QSPR model developed by Tian et al. [79], and a negative correlation was observed by Turner et al. [78].

To achieve better prediction ability and insights into the most relevant molecular features, it is important to use very large datasets and simpler systems.

### 3.2. QSPRs Derived from Ex Vivo and In Vitro Permeability Datasets

There are a high variety of experimental ex vivo and in vitro assays for the evaluation of permeability through the intestinal epithelium (see references [80,81,82] for recent reviews). The models go from the use of intestinal preparations that capture the in vivo complexity (e.g., Ussing chamber systems [83,84]) towards simple model systems focused on the non-polar center of membranes’ lipid bilayers (e.g., the PAMPA assay [85,86] or partition between water and organic solvents [87]).

The use of ex vivo assays has the advantage of being closer to the relevant biological system, with the observed permeability being affected by many factors including membrane permeation itself (by passive and/or active pathways) but also metabolism. While the effective permeability may be the relevant property that determines in vivo bioavailability, the complex interplay of the distinct steps will hinder the establishment of simple quantitative relationships, thus making it difficult to predict the bioavailability of compounds with properties outside the explored regions of chemical space. Other disadvantages include the high costs and ethical issues.

Klopman et al. [88] developed a model using experimental human intestinal absorption values obtained for a training set of 417 drugs, leading to a correlation R^2^ of 0.79 and a standard deviation σ of 12.32%, for both the training set and an external test dataset (n = 50). Up to 37 molecular descriptors were considered, with the number of H-bond donors being identified as the most relevant one, with a negative correlation with human intestinal absorption. Surprisingly, LogP was not statistically relevant as a descriptor and was not included in the final model. A slightly larger dataset (n = 648) was used by Hou et al. [89], with most entries corresponding to compounds that permeate by passive diffusion (n = 579). The best prediction model was able to predict the intestinal absorption of the compounds in the training set, with R^2^ = 0.71, and that of the test set (n = 98), with R^2^ = 0.81. The most relevant descriptors identified by the model were the polar surface area (r = −0.7), the number of H-bonds as donors (r = −0.68), LogD_6.5_ (r = 0.63), and the number of H-bonds as acceptors (r = −0.63). The authors have also considered the number of violations of Lipinski’s rule-of-five as an additional descriptor, which showed a correlation of −0.61. As observed by Klopman in the model discussed above, a poor correlation was observed for LogP (r = 0.48).

Although leading to the achievement of better correlations than observed for in vivo bioavailability, the small size of the datasets considered reduces the strength of the models obtained. This is mostly due to the limited availability of intestinal tissues from healthy donors. Furthermore, the complexity of the system is also very large, with contributions from permeation itself but also from metabolism.

On the other extreme of model systems’ complexity, partition between water and non-polar organic solvents (such as octanol) is a measure of compound hydrophobicity. This property influences several of the steps involved in compounds’ bioavailability, including its aqueous solubility, passive permeation through the lipid bilayer, and association with proteins, the latter influencing permeation through active and facilitated pathways as well as metabolism. Simple quantitative relationships between partition to non-polar solvents and drug bioavailability are therefore not anticipated and are not observed. Thus, irrespective of the practical convenience of these model systems, they do not allow for further advancements in the ability to predict drug bioavailability.

The parallel artificial membrane permeability assay (PAMPA) is an approach that has become very popular for the prediction of passive permeation. This assay is based on permeation through a porous hydrophobic filter material impregnated with a non-polar solvent and stabilized by a layer of phospholipids at the filter/water interface. The artificial membranes are implemented in 96-well plates, providing an easy and high-throughput method for measuring permeability. The presence of a thick, non-polar permeation barrier is a major disadvantage in PAMPA. While good correlations are observed between PAMPA permeability and the passive transcellular permeation of small reference compounds, this assay cannot capture the interplay of drug–membrane interactions in passive permeability through thin biomembranes. The results from this assay therefore do not go significantly beyond Lipinski’s rules and are not expected to provide insights for the design of new generations of drugs with improved bioavailability. To understand the rules of passive permeation, it is necessary to use barriers that capture the properties of biomembranes. This is being pursued by several authors with the improvement of existing liposome-based permeability assays and the development of new ones [52,90,91,92,93,94], although general-use and high-throughput approaches are still not available.

Cell monolayers are an easy-to-use in vitro model systems with properties half-way between the very complex in vivo (and ex vivo) systems and the simpler solvent-based models. They have the advantage of capturing much of the complexity of the in vivo membrane permeation processes, rather than accounting only for the interaction of the molecule with the non-polar center of the lipid bilayer [95]. The most well-characterized cell-based model for evaluating intestinal permeability is Caco-2 monolayers. These cells have been shown to spontaneously differentiate when cultured in permeable inserts for 21–30 days, which leads to the formation of cell monolayers with morphological and functional properties similar to those of small-intestine enterocytes [96,97]. The popularity of this model comes from its good compromise between relevance and simplicity [98,99]. It has been, by far, the most used cell model in pharmaceutical and academic contexts to predict the permeability of compounds across biological barriers. For this reason, a large number of *P*_app_ values for different molecules have been obtained using this model, which, in principle, could be used to derive QSPRs.

Several alternative cell-monolayer-based assays have been developed and used by the scientific community to evaluate permeability through physiological barriers. Among these, Madin-Darby Canine Kidney (MDCK) cell monolayers are of particular relevance, with the advantage of requiring only 7 days to generate a tight cell monolayer that may be used in permeability assays [95,100,101,102]. An additional advantage of this cell line is the availability of the MDCK-MDR1 variant providing an easy way to access back-transport by the efflux transporter P-glycoprotein (P-gp) [103,104,105,106,107]. Important disadvantages of this cell line lie in their origin from kidney canine cells, thus differentiating into a phenotype that is more similar to the kidney than to the intestinal epithelium [108,109,110,111]. This is manifested in a high permeability to small ions, leading to transepithelial resistance values for ionic conductance that are much lower than those observed in Caco-2 cell monolayers [112,113]. Many additional cell models are being explored to evaluate drug permeability through physiological barriers, mostly focused on the blood–brain barrier [114,115,116,117] but also on intestinal absorption [82,118]. Caco-2 cell monolayers are nevertheless the best-characterized cell model and are used most often in permeability studies [82]. In addition, the challenges regarding the consistency and accuracy of permeability data are mostly shared by all cell models. For these reasons, the results presented in this review are mostly from studies using Caco-2 monolayers.

#### 3.2.1. How Is the Performance of QSPRs in Predicting Caco-2 *P*_app_ Values

Although also presenting some complexity, in vitro cell-based systems are more focused on a single physicochemical process compared to in vivo models, which is membrane permeability. It is expected, therefore, that there will be a higher predictive ability for QSPRs developed using in vitro *P*_app_ datasets.

The first attempt to find a correlation function between *P*_app_ values measured across Caco-2 monolayers and simple molecular properties appeared in 1996. Waterbeemd et al. [119] used a dataset of 17 drugs that varied in their MW and lipophilicity, including molecules with non-ionizable groups only as well as weak acid and bases. For this dataset, a good correlation was achieved (R^2^ = 0.89) considering two molecular descriptors, where a positive correlation was obtained with MW and a negative correlation was obtained with the H-bonding potential. Of particular relevance is the unexpected positive contribution of MW to the QSPR equation, which may reflect correlations between the MW and lipophilicity for the considered dataset, which includes several structurally related compounds. However, the permeability coefficient for the drugs in the dataset did not correlate with the MW descriptor alone (R^2^ = 0.16), not even for the small homologous series of five beta-blocker drugs. One explanation for the lack of a correlation may be the narrow MW range of analyzed drugs (138–398 Da for all drugs and 249–267 Da for beta-blocker drugs).

Over the last decades, several QSPRs models for Caco-2 permeability predictions have been published, applying more sophisticated and computationally demanding modelling techniques, a wider range of descriptor types, and, most importantly, larger datasets. Table 1 presents some examples of QSPRs approaches developed during the last 10 years.

The analysis of their performances reveals large uncertainties in the predictions of *P*_app_ values of sets of molecules independent from the training set. Thus, some caution should be exercised when applying any of the models for quantitative predictions, as they can produce misleading information regarding the molecule’s ability to permeate biological membrane barriers. Although the more recently constructed QSPRs models have become more and more sophisticated and use larger and more heterogeneous databases, their predictive ability capacity has not been significantly improved relative to the models previously published. A striking observation from the QSPRs shown in Table 1 is that opposite trends are captured by different models for the same molecular descriptors. This may reflect correlations between the distinct molecular descriptors considered in the models. Also, depending on its value, a given descriptor may influence *P*_app_ differently [121]. As an example, when the compound has a low LogP, the polar surface area (PSA) has a very negative impact on *P*_app_, while very lipophilic compounds may tolerate a moderate PSA without significantly decreasing *P*_app_. That is, a small molecule may permeate fast only if its PSA is low, while molecules with an intermediate size may simultaneously have a moderate lipophilicity and PSA. The trend and the importance of the distinct molecular descriptors thus depend strongly on the region of the drug chemical space being considered.

#### 3.2.2. How Caco-2 P_app_ Data Is Selected and Compiled to Construct the QSPRs Models

The procedures related to the collection of *P*_app_ experimental data are crucial to ensuring data consistency for the development of more robust QSPRs models. Regarding the reported models in Table 1, with the exception of the dataset used by Sherer et al. [120] that comprises a huge amount of in-house/proprietary data and some public data, in all the other studies, the dataset was collected from public sources and is available online. The utilization of large public datasets implies that its collection had been made from more than one source. This way of compiling *P*_app_ data is particularly problematic because it introduces interlaboratory variability into the correlations derived from these datasets. This is due to the existence of variability in Caco-2 *P*_app_ values for the same compound when they are measured in different laboratories and sometimes even in the same laboratory. The variability factor is a recognized limitation of the Caco-2 permeability assays, and issues related with that have been addressed since 1990s [124,125,126].

In the process of selecting the *P*_app_ dataset to construct the model, some authors have explained their criteria when they were confronted with different reported *P*_app_ values for the same molecule. Wang and Chen [123] described that when the *P*_app_ values were not significantly different, their arithmetic mean was considered as the final value to be included in the dataset. When they found large differences in the *P*_app_ values, the data for these molecules were eliminated from the final dataset.

The selection of particular *P*_app_ measurements from multiple studies will tend to minimize the variability and increase the accuracy of the QSPR approach. But it also has a drawback, which is the potential bias that can be introduced into the correlations obtained. If applied to compound datasets obtained using the same procedures, the correlations tend to work well. Limitations for these models are, however, likely to arise if the biased correlations are extended to compound datasets obtained with different procedures. This precludes the use of these data to develop QSPRs that may be reliably applied [127]. The alternative is using experimental *P*_app_ values that are consistent across different research groups and available to the public in a database that represents a physicochemical permeability space as large as possible.

Sherer et al. [120] developed a QSPR model using the most extensive dataset ever reported, including over 15,700 *P*_app_ values of compounds. Unfortunately, the permeability data belong to a big pharmaceutical company and are not available to the scientific community, limiting its use for additional QSPRs studies. The literature is considered the primary source for the collection of Caco-2 *P*_app_ values. Yet, building a large-enough dataset to be used for in silico applications from literature sources requires a significant investment of time and effort. The available data are published in a large number of journal articles, and it is usually demanding to manually search and extract information, since each article needs to be considered on its own. For that reason, compilations and reviews of literature data already published are a much more convenient source of Caco-2 assay data, particularly those that were compiled by experts in the field and include primary references for all data [128].

In an attempt to assist in the construction of QSPRs by providing easy, free, and open access to the literature information, publicly available databases have been developed on the Web. The databases PerMM [129] (Permeability of Molecules across Membranes, 2019) and MolMeDB [130] (Molecules on Membranes Database, 2019) are the most recent efforts that have been devoted to the compilation of experimental Caco-2 *P*_app_ data extracted from literature studies. A comparison between the two databases is present in Table 2.

The PerMM (https://permm.phar.umich.edu/membrane_systems/9, accessed on 12 January 2024) database contains a set of 186 molecules with experimentally determined permeability coefficients for assays performed in Caco-2 monolayers. The molecules are divided into different chemical classes (organic acids, alkaloids, etc.) and groups with different ionization properties (neutral, bases, etc.). The permeability values were collected from a compilation of measurements published in a book chapter written by Alex Avdeef (2012) [131]. Published studies by this author investigating the membrane permeability of compounds are highly cited according to the platform Web of Science, and he is considered an expert in the field. The data were first collected from 55 studies reported in the literature, performed in different laboratories. Then, the collected *P*_app_ permeability values were pre-treated to correct for all non-transcellular effects by removing the contributions of the aqueous boundary layer, filter, and paracellular permeability. When the values of each permeability component could not be determined based on the original works, estimates for their values were calculated. Finally, the parameter reported for molecules in the database is the transcellular permeability coefficient (log*P*_c_) at pH 6.5 [129]. Notably, the use of this parameter and pH makes the comparison with other experimental data much more difficult. Another disadvantage is that the values reported in the book chapter and in the database are not linked to the primary literature reference. Instead, as exemplified for the drug propranolol, nine literature references are indicated. Analyzing all the references cited, it is observed that only six include permeability assays across Caco-2 monolayers, with the reported *P*_app_ values varying by almost two orders of magnitude, with a Log*P*_app_ from −5.4 to −3.7. The value considered in the database is −4.2, which is within the range of the reported values but does not correspond to any specific result nor to the average of all. In addition to the lack of information regarding the source of the data selected for inclusion in the database, the corresponding experimental conditions of the assay(s) and the corrections introduced are also not available. This significantly lowers the confidence in the dataset and limits its use to the specific conditions considered.

The MolMeDB (https://docs.molmedb.upol.cz, accessed on 12 January 2024) database contains 637 compounds with Caco-2 permeability values. The Log*P*_app_ data were obtained from in silico methodologies or experimental assays. For some compounds, such as propranolol, only data from in silico studies are reported. For some others, the reported Log*P*_app_ values include experimental and in silico data and vary by several orders of magnitude (e.g., salicylic acid with values from −3.4 to −5.5, Table 2). One positive point of this database is that all entries include the reference to the primary source of data. Also, the database can be easily downloaded to give access to the parameters in an organized manner. Although this simplifies the initial curation of data, a secondary curation is still necessary since the detailed description of the experimental conditions of the assays is not documented.

Another major problem of the two databases discussed is their relatively small size.

For the mentioned reasons, the information available in these databases cannot be straightforwardly used for the development of better QSPRs models for Caco-2 monolayer permeation.

## 4. Experimental Variability of Caco-2 *P*_app_ Values within Laboratories and between Laboratories

When aiming at a quantitative prediction of Caco-2 permeability using QSPRs approaches, the variability associated with the *P*_app_ data is one of the most important issues to be addressed. The experimental variability occurring between laboratories, and even within the same laboratory, conditions the creation of large and consistent databases of *P*_app_ values to be used for in silico works. To help understand the impact of this variability on QSPRs modelling tasks, it is important to first address its sources and magnitude.

### 4.1. Assessing the Magnitude of Variability within Laboratories

To assess the extent of variability within laboratories, Egan et al. [57] collected the mean and standard deviation (SD) of *P*_app_ values from a set of compounds assayed in five randomly selected permeability studies published in the literature [132,133,134,135,136]. The datasets included compounds with diverse physicochemical properties that permeate through distinct pathways. The coefficient of variation (%SD) was calculated for the replicate measurements of each compound in each publication. The mean of the coefficients of variation in each study varied from 5.6% to 28.3% (left side, Table 3). Although there are some differences in the internal variability in each laboratory, the coefficients of variation were small or moderate. A direct correlation between the number of replicates and the coefficients of variation may be observed, suggesting that in some of the studies, the number of replicates may be insufficient, leading to an artificially low variability.

The extent of the variability is evaluated individually for some reference compounds included in the datasets (right side, Table 3). The *P*_app_ values of the hydrophilic marker mannitol showed variations of 20 to 30% among assays performed in the same laboratory. A smaller internal variability is observed in all laboratories for propranolol (4 to 14%), while for amoxicillin, a larger variability was observed (25 to 47%). These compounds were selected due to the distinct permeation pathways, with mannitol permeating paracellularly and propranolol permeating transcellularly by passive diffusion, while amoxicillin is a substrate of the PepT1 transporter. The distinct internal variability obtained for the three compounds suggests that the paracellular pathway and transporter expression is more sensitive to small variations within a given laboratory. Yazdanian et al. [133] carried out 102 permeability assays with mannitol over 22 months in its laboratory. The observation that the variability for *P*_app_ values of mannitol is among the highest reinforces the concern that, in some cases, the small variability observed is due to an insufficient number of independent replicates.

### 4.2. Assessing the Magnitude of Variability between Laboratories

The variability in *P*_app_ values measured in different laboratories has been highlighted and discussed in several publications. Early in 1996, Artursson et al. [124] showed that the results obtained for a set of reference compounds in four different laboratories led to four different sigmodal relationships between Caco-2 *P*_app_ and the fraction absorbed in humans. The deviation obtained between the distinct laboratories reached 1.75 log*P*_app_ units, an interval larger than the width of the transition between poorly to fully absorbed drugs. This inter-laboratory variability has severe consequences in the classification of compounds according to their *P*_app_ values. More recently, Lee et al. [137] reported the quantitative differences found in the *P*_app_ values of 10 compounds determined in seven distinct laboratories, including that of the authors. The majority of compounds analyzed showed variations lower than 10-fold in the *P*_app_ values obtained across the independent laboratories. However, variations as high as 30-fold and 60-fold were found for two of the compounds in the set (propranolol and metoprolol, respectively).

**Figure 2:**
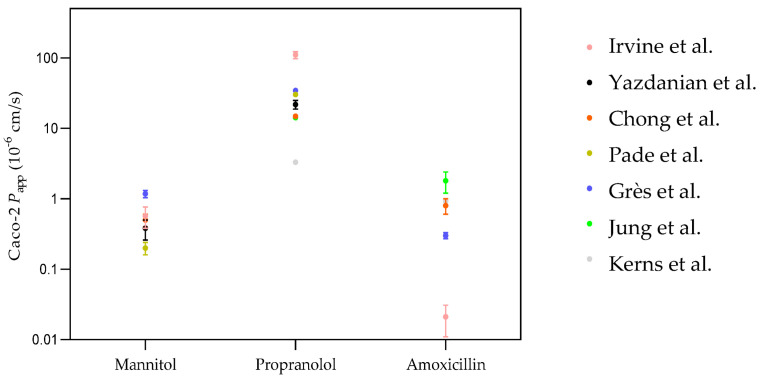
Variability in Caco-2 *P*_app_ values obtained in experiments carried out in seven different laboratories. The plot includes values for mannitol (a marker for paracellular passive permeation), propranolol (a reference drug for transcellular passive permeation), and amoxicillin (mainly transported by PepT1). The mean and SD of *P*_app_ values were taken from the studies presented in Table 3 [132,133,135,136] and other studies [138,139,140] selected from references cited in [137,141]. The values were plotted using GraphPad version 8.4.2.

The inter-laboratories variability is not limited to compounds that permeate through a specific transport route, in contrast to what was identified in the analysis within a given laboratory (Table 3). This is highlighted in the scatter plot constructed from *P*_app_ values obtained in seven distinct laboratories for three reference compounds permeating through distinct routes (Figure 2).

Interestingly, a smaller variability was observed for the paracellular marker mannitol, in contrast with the larger variability observed within each laboratory. This may reflect the use of mannitol as an internal control for the selection of properly formed Caco-2 monolayers, with those monolayers showing *P*_app_ values outside the expected range being discarded. The Log*P*_app_ values for mannitol ranged from −0.70 to 0.07, corresponding to a 6-fold variation in *P*_app_, while that of propranolol varied between 0.52 and 2.04, corresponding to a 30-fold variation. The largest inter-laboratory variability was observed for amoxicillin, with experimental values ranging from −1.68 to 0.26 (85-fold variation in *P*_app_). These results show that a direct quantitative comparison of inter-laboratory *P*_app_ values is very difficult or even impossible. To narrow the inter-laboratory variability and allow for the quantitative analysis required for the establishment of QSPRs, the sources of variability must be identified and taken into account.

## 5. Analysis of the Sources of Variability and Their Impact on *P*_app_ Values

The Caco-2 model has been the focus of several studies addressing the probable causes of variability for permeability data obtained within and between laboratories [126,142,143,144,145]. The factors encountered to explain the variability were mainly of two types: (i) heterogeneity of the Caco-2 cell line and (ii) variations in the protocols followed for cell culture and permeability assays. The most common sources of variability and their impact on permeability values are analyzed in greater depth in the following sections. To facilitate the analysis, the sources of variability for the two main phases of the Caco-2 assays, the cell culture and the permeability experiments, are analyzed separately. For each aspect, the analysis is conducted in two steps. In the first stage, a global analysis of several protocols from the literature is carried out to identify the most relevant experimental factors that may contribute to variability. In the second stage, a detailed analysis is carried out for each of the experimental variables identified in the previous step regarding their variation in the protocols and their impact on *P*_app_ values.

### 5.1. Sources of Variability Related to the Cell Culture

**Figure 3:**
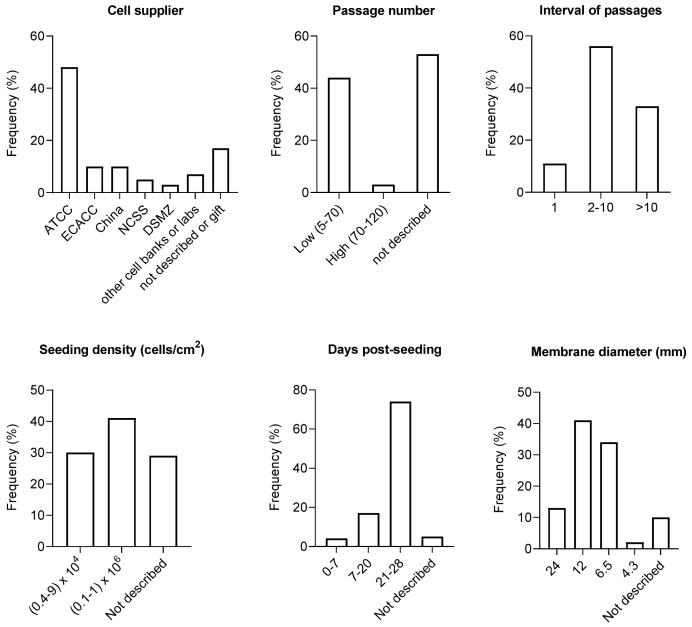
Experimental factors related to the cell culture that are potential causes of variability in Caco-2 permeability data, obtained from a survey of the literature published between 2015 and 2020 (221 papers). The distinct experimental variables and their frequency of use in the studies are shown. ATCC: American Type Culture Collection; ECACC: European Collection of Authenticated Cell Cultures—UK; NCSS: National Centre for Cell Science—India; DSMZ: German Collection of Microorganisms and Cell Cultures GmbH—Germany; China: includes several cell banks and institutes.

To better identify the differences in the methodologies followed to grow Caco-2 monolayers, a literature survey of Caco-2 permeability studies published between 2015 and 2020 was carried out, yielding a total of 221 works. These works were thoroughly reviewed to extract the information on the experimental procedures followed during the culture of Caco-2 cells pre- and post-seeding on Transwells^TM^. The outcome of this global analysis is shown in Figure 3. Based on variations in experimental conditions between protocols, it is possible to identify the most relevant factors that could be potential causes of variability (named above the graphs).

To evaluate the impact of each variable on the measured permeability, the *P*_app_ values obtained under the distinct experimental conditions are compared. A quantitative analysis can only be conducted when systematic studies were performed where the variable under evaluation is the only change in the protocol. Whenever this information existed, this analysis was performed for compounds permeating through distinct pathways. The results obtained from this systematic analysis are collected in Table 4. The effect of the variables identified in Figure 3 and Table 4 is discussed in detail in the following sub-sections and summarized in the Supplementary Material (Table S1).

#### 5.1.1. Heterogeneity of the Caco-2 Cells

The Caco-2 cell line is itself a source of variability due to its heterogeneous and unstable nature. Its cultures are characterized by the presence of different subpopulations of cells where changes may occur in the differentiation phase of the cell monolayer [156] and even in the stationary phase of growth [142]. The inherent heterogeneity of the Caco-2 populations was identified as the source of variations in cultures regarding cell morphology [146,147,157], paracellular transport across cell monolayers [146,147], enzyme expression [146], and transporters’ expression and function [147,158,159].

#### 5.1.2. Cell Source

The variability in the Caco-2 cell characteristics could be related to the cell line origin. Cell vials can be purchased from commercial suppliers (cells banks) or obtained from cultures developed in other laboratories. Cells from the ATCC supplier are the most commonly used (Figure 3).

For a comparative study, Walter and Kissel [146] grew, under identical culture conditions, Caco-2 cells obtained from two different sources, the American Type Cell Culture (ATCC) and the German Cancer Research Center (DKFZ). Dissimilarities between the cells from the two sources were found regarding several parameters, namely, their morphology, density, enzymatic activity, and paracellular permeability. Heterogeneity was also observed within the cells from each supplier. The Caco-2 monolayers obtained from cells acquired from ATCC displayed a more heterogenous morphology, exhibiting patches of cells with larger and smaller diameters. The *P*_app_ values of the paracellular marker mannitol varied by 28-fold between the two sources, whereas the *P*_app_ values of the highly permeable acetylsalicylic acid were similar. Additionally, a follow-up study by Behrens et al. [147] revealed that ATCC cell monolayers were composed of subpopulations with high expressions of the peptide transporter PepT1 and subpopulations with no expression. Contrarily, the transporter PepT1 was homogeneously distributed in monolayers prepared from cells acquired from DKFZ. In these cells, the PepT1 expression levels were about two times higher than those in monolayers prepared from ATCC cells.

#### 5.1.3. Variations in Cell Culture Protocols

The heterogeneity of Caco-2 cells and the exposure to selection pressure promoted by the respective culture conditions can give rise to the enrichment of different cell subpopulations, in a phenomenon referred to as phenotypic drift. Therefore, the maintenance of defined and consistent culture conditions has an important role in establishing reproducible experiments with Caco-2 monolayers [144]. Unfortunately, many discrepancies in culturing protocols can be identified in the literature (Figure 3). The experimental factors that vary often among the protocols from distinct laboratories include the culture media composition, the cell passage number, the density at which the cells are seeded on the inserts, the time for differentiation of the cell monolayer, and the characteristics of the membrane supports. The effects of these experimental factors are analyzed individually in the next sections.

#### 5.1.4. Culture Media Composition

The components included in the culture media can influence the phenotype and growth of Caco-2 cells, thus modulating their morphological and functional properties, such as differentiation, transporters activity, and permeability in general.

In the study of D’Souza et al. [148], Caco-2 monolayers were cultured in media containing physiological (5.5 mM) or high (25 mM) glucose concentrations. The authors reported that higher glucose concentrations significantly affected the cell’s monolayers’ integrity and permeability. The cell monolayers obtained from cells cultured at 25 mM glucose showed higher *P*_app_ values for both mannitol (an increase of 65%) and hydrocortisone (an increase of 24%), which permeate by passive paracellular and transcellular diffusion, respectively. The results for mannitol reflect a decrease in the cell monolayer tightness, further supported by a decrease in the transepithelial electrical resistance (TEER) values. This property depends on the ionic conductance of the paracellular route, and changes reflect variations in the cell monolayer integrity [160,161]. The results for hydrocortisone reflect a decrease in the barrier properties of the cells’ membrane, also supported by an increase in the membrane fluidity, as evaluated by fluorescence anisotropy. In the case of digoxin (a P-gp substrate), the *P*_app_ values increased by about 20% in both A-B and B-A directions, while the efflux ratio was not significantly affected, in agreement with a higher passive permeability and no effect on the activity of the efflux transporter. The activity of PepT1 on the transport of the peptide Gly-Sar was also shown to be influenced by the high concentration of glucose, with a decrease in the maximum transport capacity without alterations in the substrate affinity (Table 4). Collectively, the results were interpreted in light of an increased oxidative stress at higher glucose concentrations, which has been shown to influence the barrier properties of the cell membranes [162,163], the development of tight junctions [164,165], and the properties of some transporters [166].

DeMarco et al. [149] removed glutamine from the cells’ monolayer culture media, which is known to be an essential nutrient for the maintenance of intestinal mucosal integrity, and the result was an increase in the *P*_app_ values of mannitol.

Ranaldi et al. [150] observed that the *P*_app_ values of mannitol were higher in Caco-2 cells cultured with a serum-free medium as compared to a serum-supplemented medium. The deprivation of serum components in the culture medium was found to affect the maturation of tight junctions during the differentiation period, leading to an increase in the paracellular permeability of cell monolayers growing under these conditions.

Behrens at al. [153] showed that the supplementation of culture media with peptone increased the expression of PepT1 by 1.5- to 2-fold, leading to an increase in the transport of its substrate cephradine. Although this could be an expected result due to the induction of transporter expression when cells are cultured in the presence of a transporter substrate, it is relevant to note that it was not observed with the supplementation with other PepT1 substrates such as penicillin or streptomycin.

#### 5.1.5. Number of Passages

It is now well established that Caco-2 cell properties vary as the number of passages increases. The explanation for such effect has been ascribed to the heterogeneity of Caco-2 populations, with different rates of proliferation for each sub-population. As the passage number increases, an enrichment in the subpopulations with faster growth will be observed, which may lead to changes in the overall characteristics [151].

Due to the propagation of the Caco-2 cell line across laboratories worldwide, the stocks of Caco-2 cells may differ by dozens and even hundreds in terms of the number of passages. In the publications where this variable is reported, the cells have been used in permeability studies at passage numbers from 5 to 120 (Figure 3). Yu et al. [151] and Lu et al. [152] compared the properties of Caco-2 cells from early (28–36 and 35–47) and late passages (93–108 and 87–112). Briske-Anderson et al. [167] examined cells that were serially passaged 90 times from the 19 to 109 passage numbers. The three studies reported changes in morphology, proliferation, differentiation, and permeability.

The most prominent difference regarding the cell’s morphology was the detection of regions composed of multiple layers of cells for passage numbers above 87, while those where not observed for passages between 35 and 47 [152]. However, the growth of multiple cell layers may also be due to other factors since the formation of cell monolayers at passages higher than 87 has been shown by several authors [168,169,170]. Furthermore, the cells’ proliferation rates before reaching confluency were found to be higher in cells with higher passage numbers [151,152,167]. An increase in the TEER values of cells at these higher passages was also observed by all the authors. At the early stages of the monolayers culture, the increment in TEER values was explained by the faster rate of growth leading to a higher cell density for cells at later passages. Nevertheless, after the confluence was reached, cells at higher passages still displayed higher values of TEER [151,152]. At this later stage of the culture, the TEER is a reflection not only of the cell density but also of the integrity of the developed tight junctions. In agreement with this, in the study by Yu et al. [151], the increment of TEER values was accompanied by a decrease in the paracellular diffusion of mannitol. However, this was not the case in the study by Lu et al. [152] where the cell passage number increased TEER values but had no effect on mannitol permeability. [152] Conflicting evidence among studies has also been presented for the influence of the cell passage number in carrier-mediated transport. The PepT1 activity was tested with the substrate glycylsarcosine, whose *P*_app_ value remained unaltered in late and early passages [152]. Contrarily, the *P*_app_ value of the PepT1 substrate cephradine was found to diminish by fivefold in late passages in a study by Yu et al. [151] (Table 4). These studies exemplify the high variability observed in the results from this assay and point towards contributions from other factors.

Given the variation in the cell’s characteristics with the passage number, the use of the same batches of cells is recommended to avoid the selection of sub-populations and thus reduce cell-to-cell variations. This means that cells with close passage numbers should be used for related experiments. Artursson et al. [168] recommend using a window of 10 passages, between 95 and 105, to perform the permeability assays. The variability in the interval of passage numbers used in several literature studies can be seen in Figure 3. Mostly, an interval of 10 passages is followed in more than 50% of the studies.

#### 5.1.6. Seeding Density

The initial density of cells seeded on the membrane support influences the time needed to reach cell confluence, which must be attained before cell differentiation can occur. Therefore, different seeding densities may result in variations in the differentiation stage, even though the monolayers have the same age in terms of the number of days in the culture [142]. The seeding densities reported in literature studies may differ by almost three orders of magnitude, ranging from 10^3^ up to 10^6^ cells/cm^2^ (Figure 3).

Behrens et al. [153] compared the properties of cell monolayers 21 days after being seeded at low, intermediate, and high cell densities. The lowest seeding density (1 × 10^4^ cells/cm^2^) led to thinner cell monolayers, which also presented alterations in the organization of tight junctions. Multiple cell layers were observed only for the highest seeding density (1 × 10^5^ cells/cm^2^). Nevertheless, the paracellular permeability remained unaltered between the distinct seeding densities, as confirmed by the *P*_app_ values of FITC-Dextran. An identical result was obtained for the carrier-mediated transport of cephradine by PepT1, which was not affected by the seeding density, nor was the level of PepT1 expression. However, the expression levels of P-gp were significantly higher in the intermediate cell density (6 × 10^4^ cells/cm^2^). This was interpreted as a sign of decreased cell monolayer differentiation when using too high and too low cell densities.

#### 5.1.7. Days Post-Seeding on Inserts

The number of days during which Caco-2 cells are allowed to stay in the porous membrane support is a crucial parameter in cell differentiation. Already from the early studies, it has become clear that at least 21 days after seeding are required to obtain a confluent and differentiated cell monolayer for use in transport assays [96,156,171]. In fact, this is the most common procedure, which is followed in 74% of studies reported in the literature. Nevertheless, in a significant number of publications (21%), the permeability assays were performed with cell monolayers less than 21 days after seeding (Figure 3).

The time of the cell culture in the inserts was shown to influence their morphology, differentiation, tightness, and expression of transporters [153,154].

Behrens et al. [153] analyzed the cell monolayers’ properties after 7, 14, 21, and 28 days post-seeding at a density of 6 × 10^4^ cells/cm^2^. Through the visualization of the actin filaments and nucleus by confocal microscopy, the authors found significant changes in the cell morphology and cytoskeleton maturation (actin staining). During the first two weeks (days 7 and 14), the monolayers were not fully differentiated, as demonstrated by a thin and flat monolayer with weak actin staining and many small nuclei, indicating that a large number of cells are in the process of division. On the third week (day 21), they found columnar-shaped cells with a well-established brush border. The paracellular permeability was evaluated with FITC-dextran MW 4000, being low at day 7, increased at day 14, and continuously decreased for longer periods, reaching the lowest value at day 28. The expression levels of the PepT1 transporter continuously increased from day 7, reaching a maximum between days 21 and 28. Accordingly, the transport of its substrate, cephradine, also increased with the cell monolayer age. In this work, it was also shown that the P-gp expression levels reached a peak at day 21 and then declined at day 28. The effect of the day of post-seeding on P-gp expression was also evaluated by Hosoya et al. [154]. In contrast with the previous study, it is shown that P-gp expression is significant along the entire culture period of 27 days, with a maximum at day 27. However, the *P*_app_ values of the P-gp substrate cyclosporin A were enhanced in the B → A direction only after day 17, reaching a maximum at day 27. This suggests that, although present, P-gp may not be fully functional until day 17 (Table 4).

#### 5.1.8. Characteristics of the Membrane Support: Material, Coating, Diameter, and Pore Size

Different types of permeable membranes are commercially available for growing cell monolayers, presenting specific physical properties regarding the type of material, the diameter, and the size of the pores. Aimed at improving cell growth and differentiation, some authors have also proposed coating the filters with hydrogels prepared from the proteins usually found on the extracellular matrix in vivo, such as collagen, before cell seeding. The cultivation of the cells on membranes with distinct characteristics was found to influence the cells’ morphology [153], the selection of subclones [146], the proliferation rate [172], and the differentiation [146,153,155].

Polycarbonate (PC) membranes are the most frequently used, but other materials such as polyethylene terephthalate (PET) and polystyrene (PE) are also used due to some specific advantageous properties. For example, PET membranes are translucent, enabling microscopic visualizations of the cell monolayers during the culture time [144].

Behrens et al. [153] cultivated Caco-2 cells on PC, PET, and PE membranes and found that the cell morphology was significantly altered between the distinct materials. Caco-2 cells grown on PET and PE membranes formed flat monolayers, indicative of poor differentiation. Moreover, PET membranes caused differences in the microvillus structure. In contrast, cell monolayers grown on PC membranes consisted of tall columnar-shaped cells with a thick microvillus structure. No significant variations were observed between the membrane type and the expression of PepT1 and P-gp transporters. However, PET membranes showed a small stimulating effect on the P-gp expression, whereas PepT1 expression was slightly decreased [153]. Cells grown on PET membranes also displayed lower *P*_app_ values for the paracellular marker FITC-Dextran (MW 4000) than cells grown on the other membrane materials. Among them, the highest FITC-Dextran *P*_app_ value was obtained for PC membranes. Accordingly, Walter and Kissel [146] also reported a higher *P*_app_ value of mannitol when cells were cultured on PC rather than PET filters (Table 4). The decrease in paracellular permeability was explained based on an increase in the tight junction’s organization, indirectly supported by a stronger actin staining observed for cells grown on PET and PE relative to PC membranes. However, this is not direct evidence, since changes in the actin organization could be caused by the different adherence of cells to the porous support membranes [173]. Tighter interactions between the cells’ basolateral surface and the material of the porous membrane could also lead to a decrease in the access to the membrane pores and therefore to a decrease in the observed paracellular permeability. In agreement with this interpretation, increasing the space between the cell monolayer and the support membrane through the introduction of a gel matrix (e.g., collagen) was shown to lead to an increase in the observed paracellular permeability [153]. Coating has also been shown to influence the properties of the cell monolayers. Behrens et al. [153] showed that coating PC filters with rat collagen resulted in an increased expression of PepT1 and P-gp transporters and produced a significant increase in the transport of cephradine, a substrate of PepT1 (Table 4).

There are commercially available TranswellTM inserts for use with culture plates of 6, 12, and 24 wells. The majority of studies in the literature have used the 12-well format, probably to achieve a balance between the number of assays per plate and a low surface/volume ratio (Figure 3).

Markowska et al. [155] determined the *P*_app_ values of mannitol and propranolol using PC membranes with the same pore size (0.4 µm) but different membrane diameters (6.5, 12, and 24 mm, corresponding to plates with 24, 12, and 6 wells). The *P*_app_ values for both compounds had a tendency to decrease as the diameter of the membrane increased. The most significant variation was found for propranolol and larger-diameter membranes (Table 4). The authors attributed this decrease in *P*_app_ to the heterogeneous nature of Caco-2 cells, with monolayers having differences in passive transport caused by the variable development of actin rings and tight junctions. Nonetheless, it would also be interesting to evaluate the possibility of the cell monolayer being less cohesive at the membrane periphery, whose contribution is lower when larger-diameter membranes are used.

Regarding the pore size, Lechanteur et al. [172] analyzed the impact on monolayer integrity when Caco-2 cells were seeded on membranes with a pore size of 1 µm or 3 µm. The monolayers exhibited differences in their TEER values, with monolayers seeded on 3 µm membranes showing the lowest values. The visualization of the monolayers using microscopic techniques allowed for the identification of two distinct characteristics in the monolayers seeded on 3 µm membranes. These monolayers showed gaps on the apical side and also the additional presence of cells on the basolateral side of the TranswellTM, resulting in a loosely packed double cell layer. The authors conclude that Caco-2 cells were able to migrate across membranes with a pore size of 3 µm but not through the 1 µm pores. Therefore, membranes with a pore size inferior to 1 µm are recommended for permeability assays, and the most commonly used is 0.4 µm [168].

### 5.2. Sources of Variability Related to the Permeability Experiments

Large differences in the experimental settings of the permeability assay protocols are commonly observed among research groups. An experimental variable that often varies between labs is the transport media used to perform the assay, which present variations in its pH and composition, including the presence of additives and/or cosolvents. Also, the stirring rate, sampling method (replacement or transfer), number of sampling time points, and total duration of the assay can differ.

The use of different protocols may have distinct effects on the properties of the cell monolayers and on the molecules being tested (e.g., distinct ionization at the pH of the assay), which ultimately can lead to changes in *P*_app_ values for the same compound. To allow for the comparison between the results obtained by distinct laboratories, it is crucial to quantitatively evaluate the impact that each experimental variable has on the *P*_app_ results. This is performed by assessing the *P*_app_ values of compounds obtained under the distinct variables, using systematic studies where only that experimental condition is changed. The analysis of *P*_app_ values of compounds originating from the use of distinct experimental variables in permeability assay protocols is shown in Table 5. The impact of each of the sources of variability included in Table 5 is discussed in detail in the next sub-sections and summarized in the Supplementary Material (Table S2).

#### 5.2.1. Composition of the Transport Media

The aqueous medium selected for performing permeability assays serves the multiple purposes of washing the cell monolayer, preparing the test compound solution, and finally, acting as transport media in the assay [189]. Simple buffered salt solutions are usually used, such as Hanks’ Balanced Salt Solution (HBSS) [190,191,192] and phosphate-buffered saline (PBS) [193,194], although a complex cell culture medium is also used in some assays [195,196,197]. The composition of salts and glucose in buffers guarantees cell viability and the maintenance of the ionic balance at the cell membrane during the experiments [198]. However, limitations can often arise in permeability assessments due to the high polarity of aqueous buffers that prevents the solubilization of non-polar compounds. This can give rise to their immediate precipitation and the consequent turbidity of the buffer solution before being added to the donor compartment [199]. Using a cell culture medium could address this issue. However, this medium contains a large variety of components that may influence the transport of the test compounds in a way that is difficult to predict. A better approach is to supplement the ionic buffer solution with simpler and well-defined solubilizing agents. Before analyzing in detail the impact of specific solubilizing agents, it is important to systematize the distinct effects expected.

The first distinction is whether the solubilizing agent interacts with the cell monolayer (A) or not (B). Case A may lead to very distinct effects depending on the solubilizing agent and how it interacts with the cell monolayer. It may disrupt the tight junctions (A1), leading to an increase in the paracellular permeability. The solubilizing agents may partition to the cell membrane, changing its fluidity and therefore passive transcellular permeability (A2). Or, it may interact with transporters (A3)—in this case, with many possible outcomes. Competition for transport by efflux proteins is one of the most common situations, leading to an increase in *P*_app_ in the A → B direction and a decrease in *P*_app_ in the B → A direction. When the solubilizing agent does not interact directly with the cell membrane, the effects mostly depend on how the test compound was present in the solution. If large aggregates were present, the solubilizing agent will dissolve the aggregates or decrease their size, leading to an increase in the amount of the compound available to interact with the cell monolayer and, therefore, to an increase in *P*_app_ (B1). However, if the test compound was not aggregated or if the aggregates were small and dynamic, the solubilizing agent will not necessarily increase the compound availability (B2). In this case, the outcome will depend strongly on the properties of the solubilizing agent. The association of the compound with large solubilizing agents such as liposomes, large micelles, proteins, or other polymers will decrease the amount of the compound in the aqueous media and will likely decrease its permeability (B2a). However, if the solubilizing agent is a co-solvent, the effect will mostly be a decrease in the adsorption of the test compound to the apparatus, leading to an increase in *P*_app_ (B2b). The organic solvents may also lead to changes in the properties of the aqueous media, such as the viscosity or osmolarity, with the outcome in the observed *P*_app_ being case specific and difficult to rationalize.

Solubilizing agents are occasionally also added to the acceptor compartment. In this case, the most important objectives are to decrease the adsorption of the test compound to the apparatus, to decrease its retention in the cell monolayer, and to guarantee sink conditions [182]. Due to the many possible effects, changes in the *P*_app_ values cannot be easily systematized.

The impact of some typical solubilizing agents on the *P*_app_ values of compounds is presented and discussed in the sections bellow.

#### 5.2.2. Addition of Surfactants, Amphiphilic Polymers, and Co-Solvents

Bile salts, synthetic surfactants, amphiphilic polymers, and small percentages of co-solvents are usually incorporated into the transport solution of the donor compartment and occasionally also in the acceptor compartment.

The use of bile salts as solubilizers in permeability assays is a natural choice considering their presence in the small intestine, particularly during lipid digestion. Bile salts form small micelles above their critical micelle concentration, which can solubilize poorly soluble molecules (e.g., cholesterol and liposoluble vitamins), enhancing their absorption at the intestine [200]. Yamashita et al. [174] showed that the presence of 10 mM of taurocholate or cholate bile salts on the donor side had no effect on the *P*_app_ values of the lipophilic drug dexamethasone (logD_7.4_ = 2.01). In contrast, the *P*_app_ value decreased to half with the use of 10 mM of the synthetic surfactant sodium lauryl sulfate (SLS) (Table 5). This result was interpreted on the basis of a decrease in the fraction of dexamethasone available for permeation due to its association with the large SLS micelles. Nevertheless, the presence of SLS also caused a decline in TEER values, indicating that the integrity of the cell monolayer was compromised. In addition, SLS interacts efficiently with cell membranes [201], increases their fluidity [202], and has been shown to modulate P-gp activity [203]. This solubilizing agent may therefore be included as cases A1, A2, and A3, each one with different effects on *P*_app_. The overall result was a balance between all effects. The bile salts used in that study also interact with the cell membranes [28,204], influence their fluidity [204], and modulate P-gp activity [205]. However, their lower lipophilicity (Log*K*_P_ for POPC liposomes equal to 2.6 for cholic acid [28] and close to 5 for SLS [201,206]) leads to lower local concentrations in the membrane and lower membrane perturbation, resulting in no effects on the observed permeability of dexamethasone.

Saha et al. [175] measured the permeability of three poorly soluble molecules, Sch 56592, Sch-X, and Sch-Y, in the presence of 1% (*v*/*v*) of various solubilizing agents, including the amphiphilic polymers povidone and pluronic F68 and the surfactant gelucir 44/14. As observed by Yamashita et al. [174], the presence of the surfactant has an impact on many aspects. A key distinction in this study is the aggregation of test compounds in the absence of the surfactant, while in the previous study, the test compound was at a concentration below its solubility in the aqueous medium. Therefore, in addition to the effects on the cell membrane, there is also an increase in the amount of the test compound solubilized (Case B2). The solubility of Sch-X is increased by 133-fold in the presence of the surfactant and the value of *P*_app_ is increased to 165%, indicating that factor B2b dominated the overall result. A lower increase in solubility was observed in the case of Sch 56592 (12-fold) and Sch-Y (2-fold), and their *P*_app_ values decreased to 30% and 20%, respectively. This indicates that the decrease in the availability due to binding to the surfactant micelles was dominant for these test compounds (case B2a). The effect of the amphiphilic polymers was always an increase in *P*_app_, more significant for Sch-X. The overall impact of the polymers is intricate, involving various aspects, and is difficult to rationalize.

Dimethyl sulfoxide (DMSO) and ethanol are some examples of organic solvents usually incorporated as solubilizers. At concentrations below 2%, these solvents show little toxicity and lead to non-significant variations in *P*_app_ [207,208] in the case of test compounds that were already solubilized in the aqueous medium. For poorly soluble compounds, the addition of the organic solvent is expected to lead to an increase in *P*_app_ (Case B2b), although no direct support for this effect could be found in the literature. In contrast, Yamashita et al. [174] and Aungst et al. [176] observed a small decrease in *P*_app_ in the presence of 2% DMSO or dimethylacetamide, respectively. This effect points towards changes in the physical properties of transport media, such as an increase in viscosity.

#### 5.2.3. Addition of Bovine Serum Albumin (BSA)

Reaching the blood circulation, the molecule*s* are to some extent bound to proteins and lipoproteins in the plasma or are dissolved in plasma aqueous medium (unbound). The main protein responsible for the binding in plasma is human serum albumin, which binds most hydrophobic compounds. Only the unbound fraction of the molecule is free to diffuse across the endothelial membranes into the tissues, which is a limiting factor for their permeation through blood endothelia and distribution to the tissues [209]. In general, bovine serum albumin (BSA) is used for in vitro assays due to its similarity with human serum protein [210,211] and much lower price.

The addition of BSA to the transport medium of Caco-2 assays has been suggested with the purpose of overcoming four main difficulties usually found in the permeability assessment of poorly water-soluble and strongly lipophilic compounds. Depending on the problem, BSA can be added to the donor, acceptor, or both compartments. BSA is usually added to both sides to solve (i) the low solubility of the compound in the aqueous medium and/or (ii) the unspecific adsorption of the compound to the apparatus. On the other hand, BSA is added only to the acceptor side to reduce (iii) the accumulation of compounds in the cells and (iv) to guarantee sink conditions [199]. The inclusion of serum albumin in the acceptor compartment of Caco-2 permeability assays is also a natural choice that approaches the in vivo conditions. A BSA concentration of 4% (*w*/*v*) is typically used since it is the concentration of albumin when the lumen side is perfused with the blood. Some specific examples are included in Table 5 and will be discussed below.

Regarding the addition of BSA to the acceptor compartment only, Aungst et al. [176] reported that 4% (*w*/*v*) BSA leads to a marked increase in the *P*_app_ values of chlorpromazine (5.4-fold), a moderate increase for phenytoin (1.4-fold), but no effect for atenolol or warfarin. The results are correlated with the compounds’ lipophilicity, with their cLogD_7.4_ (calculated using the MarvinSketch software (version 22.9.0, http://www.chemaxon.com, accessed on 16 April 2024)) varying from 2.74 for chlorpromazine to −1.80 for atenolol, with intermediate values for phenytoin and warfarin. In another study, Krishna et al. [178] evaluated the effect of 1% DMSO or 0.5 to 4% BSA on the recovery of the very lipophilic SCH-A (LogP = 6.32). The recovery was only 40% in the presence of 1% DMSO and increased to 75% with 4% (*w*/*v*) BSA. This increase in recovery was mainly due to a decrease in the amount of SCH-A retained by the Caco-2 monolayer, which dropped from 54 to 26% when 4% BSA was added. Also, the amount adsorbed to the apparatus on the donor side decreased from 8.5 to 0.2% in the presence of 1% DMSO or 4% BSA, respectively. As expected, the increase in the amount of SCH-A quantified in the acceptor compartment leads to an increase in the calculated *P*_app_ values, fivefold higher at 4% BSA relative to 1% DMSO. A similar trend was also observed for the other lipophilic molecules SCH-B (LogP = 5.89) and progesterone (LogP = 3.87), with an increase in their *P*_app_ values of fourfold and threefold. In contrast with the strong effect in the case of very lipophilic compounds, no significant effects were observed in the case of compounds with low or moderate lipophilicity, mannitol (LogP = −2.5), SCH-E (LogP = 2.14), metoprolol (LogP = 2.15), and propranolol (LogP = 2.5) [177,178].

The inclusion of serum albumin in both the donor and acceptor compartments is also a common approach followed in Caco-2 assay protocols. Yu et al. [180] studied the effect on the permeability of curcumin, which has a very low solubility in water, when using DMSO or 4% (*w*/*v*) BSA on the donor side as solubilizing agents, while the acceptor compartment always included 4% BSA. The results showed a twofold decrease in the *P*_app_ value of curcumin when BSA was added to the donor compared to the addition of DMSO.

It is not common to find studies where BSA is added only to the donor compartment because the assays are mainly to model absorption across the intestinal epithelium (A → B direction), where serum albumin is only present on the acceptor side. However, when studying the permeability in the secretory direction (B → A), the addition of BSA to the donor compartment is a natural choice. Neuhoff et al. [177] showed that the addition of 4% BSA to the basolateral compartment decreases the *P*_app_ in the B → A direction for metaprolol and propranolol, with no effects for mannitol. Katneni et al. [179] has systematically and quantitatively studied this effect using several compounds, observing a small decrease in *P*_app_ for propranolol and a larger decrease for diazepam. In both studies, the reduction in *P*_app_ was explained by a smaller amount of the compound being available to permeate—specifically, the unbound fraction.

The studies discussed above show that the presence of BSA in the transport medium does not alter the *P*_app_ values for hydrophilic compounds [176,177,178,179]—that is, for compounds that do not significantly bind to BSA, that do not adsorb to the assay apparatus, and that are not sequestered in the cell monolayer. This result also shows that the presence of BSA does not influence the properties of the cell monolayer, which is in agreement with the absence of strong interactions between the protein and the cell membranes [212]. This stands in contrast with the effect of other commonly used solubilizing agents like surfactants, amphiphilic polymers, or organic solvents, which may interact with the cell monolayer and alter their properties.

The *P*_app_ measured for compounds that bind moderately or strongly to BSA decreases when the protein is added to the donor compartment [177,179,180], primarily because of a reduction in the fraction of the unbound compound available for permeation. When this is the sole factor at play, it is possible to estimate the *P*_app_ value in the absence of BSA by reverse calculation, considering the binding affinity for BSA [177,179]. In some studies, the decrease observed in *P*_app_ is lower than that predicted by the fraction of the unbound compound. This may reflect massive aggregation and/or significant adsorption to the apparatus in the absence of BSA in the transport medium. This is the case observed for curcumin, where the reported binding affinity is 2 × 10^5^ M^−1^ [213], leading to less than 10% of the unbound curcumin at 4% BSA. A decrease of at least 10-fold in *P*_app_ was expected, while only a 2-fold decrease in *P*_app_ was observed [180].

The use of BSA as a solubilizing agent in the donor compartment is not, therefore, necessarily a factor of variability in the *P*_app_ reported in permeability assays. Even when the *P*_app_ corrected for the unbound fraction is not reported, it may be calculated based on the observed *P*_app_, provided that the concentration of BSA and the binding affinity are known. This is another advantage of using BSA in comparison with other solubilizing agents.

When BSA is added to the donor compartment, an equally efficient binding agent should also be added to the acceptor side. Otherwise, the equilibrium with the acceptor compartment would be achieved at a very small amount of the compound transported, and sink conditions could not be guaranteed. In the studies discussed above, it was shown that adding BSA to the acceptor compartment leads to an increase in the observed *P*_app_ [176,177,178]. In this case, the *P*_app_ value to be considered is that in the presence of BSA, because in the absence of the solubilizing agent, the conditions required for the assay were not verified. Namely, that there must be (i) no significant sequestration in the cell monolayer, (ii) no significant adsorption to the apparatus, and (iii) less than 10% of the compound being transported to the acceptor compartment relative to its equilibrium concentration in this compartment [168].

#### 5.2.4. Selection of the Transport Media pH Value

The pH at the apical side of the cell monolayer constitutes a very important factor for ionizable compounds. Depending on the p*Ka*, minimal variations in the pH value can lead to significantly different fractions of ionized and unionized species and, because the passive transcellular permeability of neutral species is usually significantly larger [214], lead to large variabilities in the *P*_app_ values observed.

The permeability assays are often performed at a fixed pH of 7.4 in both the apical and basolateral compartments. To obtain this pH, the buffer medium is supplemented with 4–2-hydroxyethyl-1-piperazineethanesulfonic acid (HEPES at 10 or 25 mM). However, to mimic the conditions found in vivo across the intestinal epithelium, a pH gradient can be established across the cell monolayer by using a pH of 5.5–6.5 in the apical compartment and a pH of 7.4 in the basolateral compartment. This is particularly relevant in the case of active transport because the functionality of pH-dependent active carriers depends on the presence of a pH gradient acting as a driving force. To obtain the acidic pH, the buffer medium is usually supplemented with 2-Morpholinoethanesulfonic acid (MES–10 or 25 mM).

Yamashita et al. [174] compared the *P*_app_ values measured in the absence and presence of a pH gradient, (7.4/7.4) versus (6.0/7.4). The results revealed that dexamethasone and FITC-dextran were not affected by the pH. However, changing the apical pH from 7.4 to 6.0 increased the *P*_app_ values of weak acids (salicylic acid) and PepT1 substrates (ampicillin), while the opposite occurred in the case of weak bases (atenolol) (Table 5). The permeability results for salicylic acid and atenolol are explained based on the variation in the fraction of the neutral form of the compounds. For ampicillin, at pH 7.4, the anionic form represents 60% and the zwitterionic form represents 40%, while at pH 6, it is mainly in the zwitterionic form (94%) (calculated using the MarvinSketch software (version 22.9.0, http://www.chemaxon.com, accessed on 16 April 2024)). The higher permeability observed at pH 6 is difficult to understand in terms of variations in the fractions of the different forms, since both the anionic and the zwitterionic forms have low passive permeability. Therefore, the effect of pH points towards another mechanism of transport across Caco-2 monolayers. Ampicillin is known to be a substrate of the PepT1 transporter. The higher *P*_app_ values observed for ampicillin under the pH gradient can be explained due to its transport by the PepT1 transporter, which is driven by a transmembrane H^+^ gradient [215].

An important drawback of the use of a pH gradient in permeability assays is that it may lead to misleading results regarding the existence of active transport for compounds with weak basic or acidic groups. Neuhoff et al. [181] measured the bi-directional transport of atenolol when the apical pH was reduced from 8 to 5 and reported a decrease in *P*_app_ values in the A-B direction by a factor of eight compared to no changes in the *P*_app_ values in the B-A direction (Table 5). At the lower pH, the concentration of uncharged species decreased, resulting in a “false” efflux, even though atenolol permeates mainly by passive pathways. As expected, no difference in the transport of atenolol in both directions was found in the absence of a pH gradient.

#### 5.2.5. Concentration-Dependent Effects

There are many reasons that may lead to a concentration-dependent permeation rate. If the compound permeation is mediated by a transporter in the membrane, an increase in concentration is expected to lead to a decrease in *P*_app_ due to the saturation of the transporter [185,216,217,218,219,220], the magnitude of the effect depending on the density of the transporter in the membrane and the compound affinity (concentration at half saturation). However, the concentration effects observed for the permeation of drugs are usually in the opposite direction, with *P*_app_ increasing with the concentration of the permeating compound (entries for chlorpromazine and quinidine in Table 5) [183,185]. This is because the active transport occurs in the direction opposite to the permeation being measured, corresponding to back transport by efflux proteins. As the drug concentration increases, the influx rate by passive permeation increases linearly but the efflux rate increases sub-linearly if protein saturation is approached, leading to an increase in net-transport. An increase in net-transport is also observed in the presence of other substrates or inhibitors of the efflux proteins. This situation is exemplified by the two digoxin entries in Table 5, with the transport of digoxin in the A → B direction increasing in the presence of clarithromycin and cyclosporin A [186]. An opposite effect is observed for transport in the B → A direction due to a decrease in the rate of active efflux. Drug–drug interference may be particularly problematic in permeability assays where cocktails of different compounds are used to increase the assay throughput (e.g., [219]). Depending on the transport pathway, different stereoisomers may also permeate at different rates and with distinct concentration effects, as exemplified in Table 5 for Propranolol. Although not shown in the table, the value of *P*_app_ in the B → A direction was also different for both stereoisomers. The complex behavior observed was interpreted as the stereospecific activity of both influx and efflux transport proteins [184].

Although the effects of the concentration are usually attributed to the saturation of protein transporters, there is extensive evidence that concentration effects are observed for passive permeation as well. These may result from changes in the membrane physico-chemical properties due to the presence of the test compound or other components in the transport medium, namely, the membrane charge and fluidity [28,221,222,223]. Sterols are an example of compounds with complex effects on the membrane properties. At low-to-intermediate concentrations, they decrease the membrane fluidity, leading to a decrease in permeability [12,224,225,226,227]. However, at very high concentrations, sterols established weaker interactions with the membrane and were able to permeate faster (e.g., DHE in Table 5) [182,228]. In cell-monolayer assays, the solute concentration required to generate significant effects on the property of the membrane is unexpectedly low. This is due to the very small amount of the cell membrane present in the assay, a few nmol in a 1.12 cm^2^ insert [182]. A total concentration of the test compound equal to 10 µM corresponds to 4 nmol, similar to the amount of lipids from the cell plasma membrane. Thus, depending on the compound membrane affinity, high local concentrations may be achieved even for compound concentrations in the micro molar range.

Finally, a common situation of concentration-dependent permeability is related with cell toxicity or effects on monolayer integrity, usually leading to an increase in permeability. These effects may be due to the compound itself or to other compounds present in the transport media (see, e.g., references [229]). These effects should be avoided through the prior evaluation of cell viability tests to guide the selection of the concentrations of the test compound and the additives used in the transport medium, as recommended in all technical protocols proposed (e.g., [168]).

#### 5.2.6. Unstirred Water Layer (UWL) and Stirring Conditions

Adjacent to the cell monolayer, there is an unstirred layer of water (UWL) that acts as an additional barrier to the permeation of compounds through the cell monolayer. For fast-permeating compounds (low resistance to transport through the cell monolayer), this additional barrier leads to a strong decrease in the overall *P*_app_ (a strong increase in the overall resistance), while for compounds that permeate very slowly, the increase in the overall resistance may be negligible. The lipophilicity of the test compound is another factor that is a determinant for the effect of the UWL on the observed *P*_app_. This is due to the depletion of the compound in the aqueous media close to the membrane caused by the extensive membrane partition of very lipophilic compounds and the inefficient diffusion of the compound from the bulk aqueous medium in the absence of agitation.

A small UWL is present in the intestinal epithelium in vivo [230], but in in vitro Transwell^TM^ inserts, the UWL can be remarkably thick. The apparent UWL thickness was estimated to be 1544 µm [187], whereas the cell monolayer is generally 17 to 30 µm thick [96]. Keeping the Transwell^TM^ plates under stirring during the transport experiments reduces the UWL thickness. Appling an orbital agitation at 1090 rpm lowered the apparent thickness of the UWL to 128 µm [187]. However, stirring can also cause cell detachment from the porous membrane, compromising the cell monolayer integrity. The speed should therefore be kept as high as possible, but without affecting the cell monolayer integrity.

Conducting permeability assays at various stirring rates allows for the determination of the impact of the distinct UWL thickness on the *P*_app_ values for the test compounds. Artursson et al. [187] reported a variation in the *P*_app_ values of testosterone between 36 and 101 (10^−6^ cm/s) when the applied stirring rate changed from 0 to 1090 rpm, respectively. However, there was no significant effect of agitation on the *P*_app_ value for mannitol, indicating that the monolayer integrity was not compromised. The effect on testosterone *P*_app_ but not on mannitol reflects the higher effect of the UWL in the case of fast-permeating compounds. Similar results were obtained by Korjamo et al. [188], where the *P*_app_ values of propranolol and of verapamil increased threefold when the stirring rate changed from 250 to 420 rpm. In this study, the effect of stirring was evaluated for both the A → B and B → A transport directions. It should be noted that the increase in *P*_app_ was greater for transport in the A → B direction due to the more effective mixing in this compartment. The use of very high stirring rates could therefore lead to incorrect interpretations regarding the contribution of active transport.

#### 5.2.7. Some Additional Considerations

Although not yet properly addressed in the literature, the sampling method used in the permeability assay may also lead to variability in the *P*_app_ values obtained. Variations in the procedure include different time intervals and sampling using the transfer or replacement approach. We have recently shown that for Lucifer Yellow (LY), which permeates slowly through the paracellular pathway, a significant decrease in *P*_app_ is obtained as the sampling interval is increased from 10 min to two hours using the transfer approach [182,231]. This was interpreted on the basis that a significant amount of LY reaches the acceptor compartment during the transfer procedure, which contributes more significantly for smaller sampling intervals [231]. The transport of LY across the cell monolayer during the transfer of the insert is likely due to the imposition of an unbalanced hydrostatic pressure on the cell monolayer. This effect is not overcome by using the replacement approach because the cell monolayer is also subject to an unbalanced hydrostatic pressure during the time needed to remove the sample volume from the acceptor compartment and replace it with fresh transport media.

Another important factor that may lead to inconsistences between *P*_app_ values across datasets is related with data analysis. The values reported are usually the average *P*_app_, calculated from the individual results obtained assuming a Normal distribution for this variable. However, *P*_app_ is expected to follow a LogNormal distribution [232], and this has in fact been shown by us when analyzing large datasets for LY *P*_app_ [231]. The average value of *P*_app_ should therefore be calculated from the average Log*P*_app_, and the uncertainty should be expressed as confidence intervals. Not doing so will lead to an incorrect calculation of the average value. It may lead to an incorrect identification of outliers, which will introduce additional bias in the reported *P*_app_.

## 6. Strategies for Reducing the Variability of Caco-2 Permeability Data

Any expectation of obtaining a large dataset of Caco-2 *P*_app_ values that have been measured using the same cell population and experimental conditions is presently unrealistic. This will involve the production of enormous new amounts of *P*_app_ data by the same researcher and laboratory. But can something be done to allow different researchers to obtain consistent data and to use the abundant *P*_app_ data already existing in the literature? Motivated by the objective to improve the consistency of available *P*_app_ values, a few studies have evaluated possible solutions for overcoming the variability problem.

The standardization of Caco-2 assay procedures is imperative to achieving a reduction in the number of experimental conditions that are different among the studies. With the objective of developing a uniform/common methodology, attempts were made to create technical protocols that can be easily followed by the scientific community [161,168,233,234]. Of all of them, the protocol defined by Hubatsch et al. in 2007 [168] is the most cited, accounting for more than 900 citations. It was published in the journal *Nature Protocols* and contains, in detail, all the procedures necessary for evaluating the permeability of compounds through Caco-2 monolayers, including some suggestions and troubleshooting.

The normalization of the *P*_app_ values of a test compound relative to a reference compound was followed by some authors before assembling the various datasets that were compiled from different laboratories into a single larger dataset [141,235]. A reduction in the overall inter-laboratory variability was achieved, but the variability was still high regardless of the normalization.

When the experimental conditions of the assays generating a given *P*_app_ value are well described, it becomes valuable to attempt to convert these *P*_app_ values to those that would be obtained under standard conditions. To determine the feasibility of this standardization approach, a *P*_app_ dataset should be closely examined to search for common compounds that have been analyzed under various conditions. This will allow for a group analysis and the aggregation of compounds in datasets to distinguish the effects on *P*_app_ values based on their distinct properties (passive permeation, active transport). After compiling a larger dataset with many experimental parameters, one should search for QSPRs models to convert the results to a standard condition. Further studies aimed at understanding the possibility of the conversion of *P*_app_ values between different experimental conditions need to be conducted to validate this approach.

Although there are many sources of variability, the cell batch and the culture procedures pre- and post-seeding the cells in the Transwell^TM^ inserts are the major contributors to intrinsic variability. To reduce this variability, one possible approach is to use the same batch of Caco-2 cells for assaying a set of compounds. This can be achieved by re-using the Caco-2 monolayers in multiple permeability assays. We have recently developed a methodology for allowing the re-use while following the procedure proposed in the reference protocol. Permeability assays were performed sequentially on days 22, 25, and 28 after cell seeding, with an incubation period of two full days with culture media between each permeability assay. Consistent *P*_app_ values were obtained for several reference compounds that permeate by passive pathways (paracellular and transcellular), supporting the maintenance of cell monolayer integrity and membrane properties [231,236]. The results obtained showed that the variability associated with the re-use was lower than that obtained when performing the assay with a new batch of cells. In addition to triplicating the throughput of the assay, the re-use of Caco-2 monolayers is also fully aligned with the 3 Rs principles, as it reduces the resources needed for assays by using an already implemented cell monolayer.

**Figure 4:**
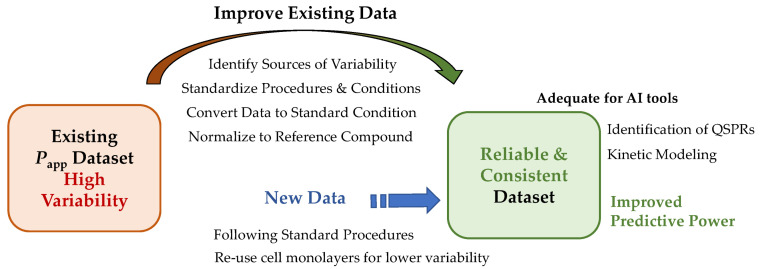
Schematic representation of strategies for reducing *P*_app_ variability and creating consistent datasets to be used in QSPR development.

These possible approaches to reducing the *P*_app_ variability and enabling the generation of large and consistent datasets are schematically presented in Figure 4.

## 7. Conclusions

The development of highly predictive and widely applicable models for predicting permeation through biological barriers is a difficult task, or even impossible, before serious efforts are made to generate consistent data. Instead of attempting the impossible task of obtaining large amounts of new data, all under the same conditions, it is more feasible to improve the data collected from the literature and/or databases. For this, it is crucial that the experimental parameters used to perform the permeability studies are thoroughly documented when compiling the *P*_app_ values from the literature and databases. Studies lacking detailed experimental conditions, such as the passage number and seeding density, should not be included in the dataset construction. Models built on these data are able to provide only qualitative insights regarding the molecules used in the study, rather than contributing to a robust, quantitative model.

In addition, the expected accuracy of QSPRs models also depends on the property being modelled. Specifically, it is important whether it is a simple process or whether it results from multiple processes. While precise QSPRs are anticipated for relatively simple processes such as the partitioning of small molecules into membranes, this is less straightforward in the case of membrane permeability. This arises because permeability can occur according to several mechanisms, each one with several steps, and depending differently on the distinct molecular descriptors. For instance, in the case of passive permeation, an increase in LogP is expected to increase the membrane affinity, but the effect on permeability depends on the rate limiting step in the permeation process. If translocation through the membrane non-polar center is the rate-limiting step, an increase in permeability is expected, but a decrease in permeability will occur if the rate-limiting step is desorption from the membrane into the aqueous medium [62,237]. In this respect, simple QSPRs are not expected for drug bioavailability in vivo, or even for permeability through complex ex vivo or in vitro systems. The approach must be to identify QSPRs from simpler model systems and use kinetic modelling to optimize the behavior in systems with increased complexity.

At all stages of system complexity, large datasets with accurate and reliable values for the evaluated property are needed. This is the first and most important step for achieving the much needed predictive power.

## Figures and Tables

**Table 1:** Overview of some examples of in silico contributions to the quantitative prediction of *P*_app_ values across Caco-2 monolayers. The analyzed research studies were published between 2012 and 2022. The information regarding the dataset sizes, the range of *P*_app_ values of the compounds included in datasets, and the relevant procedures related to their collection is indicated for each study. The predictive performance of the best model obtained in each study is reported for the test set and when applied for an external test set. The most relevant molecular descriptors and their contributions are also summarized.

Prediction Model	Data Collection				Performance ^a^	
Author, Year	Size of Training Set (tr) Test Set (t) External Test Set (ext-t)	Log*P*_app_ Range	Available Online and Free	Data Sources	Best Model	Most Important Molecular Descriptors (Correlation)
Sherer et al. [120] 2012	tr: 15,791 t: 1536 ext-t: 313	[−7.5; −4.3]	no	In-house ^b^ literature publications	R^2^_t_: 0.52 RMSE_t_: 0.20 R^2^_ext-t_: 0.41 RMSE_ext-t_: 0.91	LogP (−) Polar surface area (−) H-bond potential (−) Aqueous solubility (+)
Singh et al. [121] 2015	Tr: 508 t: 70 ext-t: 100	[−7.8; −3.4]	yes	>250 literature publications	RMSE_test_: 0.27 RMSE_ext-t_: 0.24	Polar surface area (−) LogP (+) Aqueous solubility (−)
Wang et al. [122] 2016	tr: 1017 t: 255	[−7.8; −3.5]	yes	2 public databases ^c^ 23 literature publications	R^2^_test_: 0.81 RMSE_test_: 0.31 R^2^_ext-t_: 0.75 RMSE_ext-t_: 0.36	H-bond donors (− −) Polar volume (− −)
Wang and Chen [123] 2020	tr: 1458 t: 369	[−7.9; −3.7]	yes	1 public database ^d^ Literature publications of Wang et al. 2016	R^2^_test_: 0.76 RMSE_test_: 0.39	H-bond potential (− −)

^a^ The R^2^ and RMSE (root-mean-squared error) were the metrics used to evaluate the performance of each model. The symbols + and − represent a positive or negative contribution of a specific molecular descriptor, respectively. The symbol − − indicates that the negative contribution was superior to 50%. ^b^ Proprietary *P*_app_ data were obtained across different cell lines in the pharmaceutical Merck databases. The majority of *P*_app_ data were from the LLC-PK1 cell line, which is a renal epithelial cell line derived from porcine kidney cells. The external test set includes only Caco-2 *P*_app_ values gathered from the literature. ^c^ ChEMBL and OCHEM. ^d^ ChEMBL

**Table 2:** Comparison of two online databases containing experimental data of drug permeability across Caco-2 monolayers: PerMM and MolMeDB. The databases are compared relative to the amount of data, the parameter chosen to report permeability, the sources from which the data were collected, and the values reported for the two reference drugs. The levels of organization and facility in the interpretation of the databases are also evaluated.

Database	Number of Compounds	Parameter Reported	Data Collection				Include Primary Reference	Organization Level after Download	Interpretation Level
			Experimental	In Silico	Salicylic Acid	Propranolol			
PerMM [129] 2019	186	Log*P*_c_^a^	✓		−4.57	−4.20		medium	hard
MolMeDB [130] 2019	637	Log*P*_app_^b^	✓		−5.47		✓	good	easy
			✓		−4.89				
			✓		−4.66				
			✓		−4.78				
			✓		−4.73				
				✓	−4.66				
				✓	−4.23	−4.60			
				✓	−3.39	−4.45			

^a^ Log*P*_c_ is the transcellular permeability at pH 6.5. It is extracted from Log*P*_app_ values obtained in the assays by removing the contributions of the aqueous boundary layer, support filter, and paracellular permeability from the *P*_app_ term. ^b^ parameter reported in the specific reference at the condition followed.

**Table 3:** Magnitude of the variability in Caco-2 *P*_app_ values obtained in experiments carried out in the same laboratory. The five studies analyzed were selected from Egan et al. [57]. Coefficient of variation relative to the mean (%SD) for replicates of *P*_app_ values obtained within a given laboratory. The variability is shown for the entire dataset and for some selected reference compounds.

Study	All Compounds			Mannitol		Propranolol		Amoxicillin	
	Number	Replicates per Compound	%SD ^b^	n	%SD	n	%SD	n	%SD
Irvine et al. [132]	55	6	28.3	6	33.3	6	11.8	6	47.6
Yazdanian et al. [133]	51	3–9 ^a^	12.7	102	31.6	6	14.2		
Chong et al. [135]	10	3	10.3	3	20.0	3	6.8	3	25.0
Pade et al. [136]	9	3	5.6	12	20.0	3	4.0		

^a^ with the exception of mannitol, which included 102 assays performed over 22 months. ^b^ %SD = 100 × standard deviation/mean.

**Table 4:** Analysis of the impact of several sources of variability related with cell culture protocols (pre- and post-seeding in the Transwell^TM^ inserts) on the *P*_app_ values across Caco-2 monolayers. For each variable evaluated, the permeability assays were carried out by the same research group, and that experimental condition was the only change in the protocol.

Compound ^a^	*P*_app_ (10^−6^ cm/s) A → B				Reference
	**Cell Supplier**				
	ATCC		DKFZ ^b^		
**Mannitol**	**5.23 ± 0.24**		**0.187 ± 0.007**		Walter and Kissel [146]
Acetylsalicylic acid	20.6 ± 0.7		22.7 ± 1.3		Walter and Kissel [146]
***Cephradine***	1.36		1.45		Behrens et al. [147]
	**Culture media composition**				
	5.5 mM Glucose	25 mM Glucose	−glutamine	+0.6 mM	
**Mannitol**	**0.43 ± 0.05**	**0.71 ± 0.01**			D’Souza et al. [148]
			**3.9**	**1.0**	DeMarco et al. [149]
			−serum	+10% (*v*/*v*)	
			**3.0**	**0.87 ± 0.16**	Ranaldi et al. [150]
Hydrocortisone	**20.4 ± 0.64**	**25.2 ± 0.25**			D’Souza et al. [148]
***Digoxin***	**0.99 ± 0.05**	**1.27 ± 0.05**			D’Souza et al. [148]
	**Passage number**				
	Low (28–47)		High (93–112)		
**Mannitol**	**3.4**		**2.2**		Yu et al. [151]
	5		5		Lu et al. [152]
Hydrocortisone	40		50		Lu et al. [152]
Progesterone	650		900		Yu et al. [151]
***Cephradine***	**9.49 ± 0.47**		**2.05 ± 0.01**		Yu et al. [151]
***Glycylsarcosine***	8		9		Lu et al. [152]
	**Seeding density (cells/cm^2^)**				
	Low (1 × 10^4^)	Intermediate (6 × 10^4^)		High (1 × 10^5^)	
**FITC-dextran (Mw 4000)**	0.07	0.05		0.05	Behrens et al. [153]
***Cephradine***	1.6	2.1		2.5	
	**Day post-seeding**				
	7	14	21	28	
**FITC-dextran (Mw 4000)**	0.05	**0.25**	0.04	**0.02**	Behrens et al. [153]
***Cephradine***	**1.1**	1.3	2.03 ± 0.6	1.8	Behrens et al. [153]
***Cyclosporin*** **^c^**	8	9	11	**18**	Hosoya et al. [154]
	**Membrane material and coating**				
	PC	PE	PET	PC + Collagen	
**FITC-dextran (Mw 4000)**	0.03	0.022 ± 0.0066	**0.012 ± 0.0038**	0.05	Behrens et al. [153]
**Mannitol**	22.1 ± 0.09		7.44 ± 0.33		Walter and Kissel [146]
***Cephradine***	2.0	0.75	**0.25**	**3.79 ± 1.48**	Behrens et al. [153]
	**Membrane diameter in mm** (plate type)				
	6.5 (24-well)	12 (12-well)		24 (6-well)	
**Mannitol**	**1.4 ± 0.2**	**1.3 ± 0.1**		**0.90 ± 0.1**	Markowska et al. [155]
Propranolol	**38.9 ± 1.9**	**36.6 ± 0.9**		**24.2 ± 0.3**	

The experimental variable being evaluated is highlighted in gray ^a^ Compounds shown in non-bold and non-italic permeate by passive diffusion, mostly transcellular. Compounds in Bold permeate through the paracellular pathway. Compounds in Bold italic are substrates of transporters (peptide transporter PepT1 or P-gp). The numbers in bold indicate statistically significant differences with at least one of the conditions in the variable analyzed, from the statistical analysis performed in the respective reference. ^b^ DKFZ: German Cancer Research Center. ^c^ *P*_app_ in B → A direction.

**Table 5:** Analysis of the impact of different protocols for the permeability assays on the *P*_app_ values of reference compounds across Caco-2 monolayers. For each source of variability evaluated, the permeability assays were carried out by the same research group, where only that experimental condition is varied.

Compound ^a^	*P*_app_ (10^−6^ cm/s) A → B										Reference
	**Addition of bile salts, surfactants, and co-solvents on the donor side**										
	10 mM taurocholate		10 mM cholate		10 mM SLS ^b^		2% (*v*/*v*) DMSO		2% (*v*/*v*) ethanol		
	−	+	−	+	−	+	−	+	−	+	
Dexamethasone	1.7	2.1	1.4	1.4	**1.6**	**1.0**	**1.4**	**1.0**	1.4	1.4	Yamashita et al. [174]
		1% (*v*/*v*) povidone			1% (*v*/*v*) pluronic F68				1% (*v*/*v*) gelucir 44/14		
	−	+			+				**+**		Saha et al. [175]
Sch 56592 ^c^	**100**	**140**			115				**30**		
Sch-X ^c^	**100**	135			**150**				**165**		
Sch-Y ^c^	**100**	105			**135**				**20**		
	**Addition of BSA % (*w*/*v*) on the acceptor side**										
	0		0.5		1		2		4		
**Atenolol**	0.68 ± 0.04								0.53 ± 0.04		Aungst et al. [176]
Warfarin	28.2 ± 1.5								33.1 ± 0.5		
Chlorpromazine	**9.1 ± 1.7**								**46.5 ± 2.0**		
Phenytoin	**15.4 ± 0.3**								**21.5 ± 1.2**		
**Mannitol**	0.3								0.3		Neuhoff et al. [177]
	1		2		2		2		1		Krishna et al. [178]
SCH-A ^d^	**4**		**11**		**15**		**18**		**19**		
Propranolol	8								9		
	**110**								**150**		Neuhoff et al. [177]
Metoprolol	**100**								**110**		
Progesterone	**8.2**								**22.6**		Krishna et al. [178]
SCH-B ^d^	**2.2**								**9.4**		
SCH-E ^d^	11								10		
***Digoxin***	3								4		Neuhoff et al. [177]
	**Addition of BSA % (*w*/*v*) on the donor side**										
	0		0.1		0.5		1		4		
**Mannitol**	0.3								0.3		Neuhoff et al. [177]
Metoprolol	100 ^e^								90 ^d^		
Propranolol	**100 ** ^e^								**60** ^d^		
	**36**		35		32		**28**				Katneni et al. [179]
Diazepam	**53**		46		**29**		**21**				
	**Addition of BSA % (*w*/*v*) on the donor and acceptor sides**										
	1% (*v*/*v*) DMSO/4								4/4		
Curcumin	**7.1 ± 0.6**								**3.5 ± 0.3**		Yu at al. [180]
	**pH (apical/basolateral)**										
	5.0/7.4		6.0/7.4			7.4/7.4			8.0/7.4		
**FITC-dextran**			0.006 ± 0.001			0.003 ± 0.000					Yamashita et al. [174]
**Atenolol**			**0.19 ± 0.02**			**0.40 ± 0.01**					
	**0.30 ± 0.08**					**1.26 ± 0.08**			**2.31 ± 0.09**		Neuhoff et al. [181]
Dexamethasone			12.3 ± 0.2			12.1 ± 0.5					Yamashita et al. [174]
Salicylic acid			**87.5 ± 4.8**			**3.35 ± 0.12**					
*Ampicillin*			**0.17 ± 0.01**			**0.081 ± 0.001**					
	**Concentration (µM)**										
	10			50			100		150		
Dehydroergosterol	<0.001			0.0017			0.026				Pires et al. [182]
Chlorpromazine				10.2 ± 1.6			13.3 ± 1.5		15.5 ± 1.8		Broeders et al. [183]
Propranolol (R/S)	2.5			9			10				Wang et al. [184]
	4 ^e^			7 ^e^			8.6 ^e^				
	0.1			1			10		100		
***Quinidine***				0.1			0.4		1.6		Riede et al. [185]
***Digoxin+clarithromycin ^f^***				1			2.4		4.6		Kishimoto et al. [186]
				18 ^e^			15 ^e^		6.3 ^e^		
***Digoxin+cyclosporine A ^f^***	0.7			1.7			2.4				
	11 ^e^			6 ^e^			3.6 ^e^				
	**Stirring rate**										
	0			Low (135 rpm)				High (1090 rpm)			
**Mannitol**				0.22 ± 0.08				0.26 ± 0.11			Artursson et al. [187]
Testosterone	**35.7 ± 3.3**			**51.8 ± 7.9**				**100.8 ± 7.9**			
				Low (250 rpm)				High (420 rpm)			
Propranolol				**75 ± 10**				**221 ± 13**			Korjamo et al. [188]
***Verapamil***				**57 ± 6**				**174 ± 15**			
				**64 ± 14 ^e^**				**140 ± 11 ^e^**			

Each of the evaluated variables is highlighted in gray ^a^ Compounds shown in non-bold and non-italic permeate by passive diffusion, mostly transcellular. Compounds in Bold permeate through the paracellular pathway. Compounds in Bold italic are substrates of transporters (peptide transporter PepT1 or P-gp). The numbers in bold indicate statistically significant differences with at least one of the conditions in the variable analyzed from the statistical analysis performed in the respective reference. ^b^ SLS: sodium lauryl sulfate; ^c^ Sch 56592 LogP = 2.4, Sch-X LogP not determined, Sch-Y LogP = 4.0. The values reported correspond to the amount of the compound transported per time and are expressed as a percentage in relation to transport in the absence of surfactants (100%). ^d^ SCH-A LogP = 6.32, SCH-B LogP = 5.89, SCH-E LogP = 2.18. ^e^ *P*_app_ in the B → A direction. ^f^ Digoxin *P*_app_ in the presence of different concentrations of clarithromycin or cyclosporin A.

## Data Availability

No new data were created or analyzed in this study. Data sharing is not applicable to this article.

## Supplementary Materials

The following supporting information can be downloaded at: https://www.mdpi.com/article/10.3390/membranes14070157/s1, Table S1. Summary of the usual effect on the observed Papp for several sources of variability related to the cell culture protocols (pre- and post-seeding in the TranswellTM inserts); Table S2. Summary of the usual effect on the observed Papp for several sources of variability related to the permeability assays protocols.

## References

[1] Borah P., Hazarika S., Deka S., Venugopala K.N., Nair A.B., Attimarad M., Sreeharsha N., Mailavaram R.P. (2020). Application of Advanced Technologies in Natural Product Research: A Review with Special Emphasis on ADMET Profiling. Curr. Drug Metab..

[2] Di L., Artursson P., Avdeef A., Benet L.Z., Houston J.B., Kansy M., Kerns E.H., Lennernas H., Smith D.A., Sugano K. (2020). The Critical Role of Passive Permeability in Designing Successful Drugs. Chemmedchem.

[3] Stenberg P., Bergstrom C.A.S., Luthman K., Artursson P. (2002). Theoretical predictions of drug absorption in drug discovery and development. Clin. Pharmacokinet..

[4] Sugano K., Kansy M., Artursson P., Avdeef A., Bendels S., Di L., Ecker G.F., Faller B., Fischer H., Gerebtzoff G. (2010). Coexistence of passive and carrier-mediated processes in drug transport. Nat. Rev. Drug Discov..

[5] Samanta P., Wang Y., Fuladi S., Zou J., Li Y., Shen L., Weber C., Khalili-Araghi F. (2018). Molecular determination of claudin-15 organization and channel selectivity. J. Gen. Physiol..

[6] Lingaraju A., Long T.M., Wang Y., Austin J.R., Turner J.R. (2015). Conceptual barriers to understanding physical barriers. Semin. Cell Dev. Biol..

[7] Günzel D., Yu A.S. (2013). Claudins and the modulation of tight junction permeability. Physiol. Rev..

[8] Van Itallie C.M., Fanning A.S., Anderson J.M. (2003). Reversal of charge selectivity in cation or anion-selective epithelial lines by expression of different claudins. Am. J. Physiol. Ren. Physiol..

[9] Pauletti G.M., Okumu F.W., Borchardt R.T. (1997). Effect of size and charge on the passive diffusion of peptides across Caco-2 cell monolayers via the paracellular pathway. Pharm. Res..

[10] Adson A., Raub T.J., Burton P.S., Barsuhn C.L., Hilgers A.R., Audus K.L., Ho N.F.H. (1994). Quantitative Approaches to Delineate Paracellular Diffusion in Cultured Epithelial-Cell Monolayers. J. Pharm. Sci..

[11] Mazzanti L., Ha-Duong T. (2023). Understanding Passive Membrane Permeation of Peptides: Physical Models and Sampling Methods Compared. Int. J. Mol. Sci..

[12] Filipe H.A.L., Loura L.M.S., Moreno M.J. (2023). Permeation of a Homologous Series of NBD-Labeled Fatty Amines through Lipid Bilayers: A Molecular Dynamics Study. Membranes.

[13] Benmameri M., Chantemargue B., Humeau A., Trouillas P., Fabre G. (2023). MemCross: Accelerated Weight Histogram method to assess membrane permeability. Biochim. Biophys. Acta. Biomembr..

[14] Venable R.M., Krämer A., Pastor R.W. (2019). Molecular Dynamics Simulations of Membrane Permeability. Chem. Rev..

[15] Hannesschlaeger C., Horner A., Pohl P. (2019). Intrinsic Membrane Permeability to Small Molecules. Chem. Rev..

[16] Missner A., Pohl P. (2009). 110 Years of the Meyer-Overton Rule: Predicting Membrane Permeability of Gases and Other Small Compounds. Chemphyschem A Eur. J. Chem. Phys. Phys. Chem..

[17] Krämer S.D., Lombardi D., Primorac A., Thomae A.V., Wunderli-Allenspach H. (2009). Lipid-Bilayer Permeation of Drug-Like Compounds. Chem. Biodivers..

[18] Lipinski C.A. (2016). Rule of five in 2015 and beyond: Target and ligand structural limitations, ligand chemistry structure and drug discovery project decisions. Adv. Drug Deliv. Rev..

[19] Ermondi G., Jimenez D.G., Rossi Sebastiano M., Kihlberg J., Caron G. (2023). Conformational Sampling Deciphers the Chameleonic Properties of a VHL-Based Degrader. Pharmaceutics.

[20] David L., Wenlock M., Barton P., Ritzén A. (2021). Prediction of Chameleonic Efficiency. ChemMedChem.

[21] Matsson P., Doak B.C., Over B., Kihlberg J. (2016). Cell permeability beyond the rule of 5. Adv. Drug Deliv. Rev..

[22] Leung S.S., Sindhikara D., Jacobson M.P. (2016). Simple Predictive Models of Passive Membrane Permeability Incorporating Size-Dependent Membrane-Water Partition. J. Chem. Inf. Model..

[23] Guimarães C.R., Mathiowetz A.M., Shalaeva M., Goetz G., Liras S. (2012). Use of 3D properties to characterize beyond rule-of-5 property space for passive permeation. J. Chem. Inf. Model..

[24] Sugita M., Sugiyama S., Fujie T., Yoshikawa Y., Yanagisawa K., Ohue M., Akiyama Y. (2021). Large-Scale Membrane Permeability Prediction of Cyclic Peptides Crossing a Lipid Bilayer Based on Enhanced Sampling Molecular Dynamics Simulations. J. Chem. Inf. Model..

[25] Neves M.C., Filipe H.A.L., Reis R.L., Prates Ramalho J.P., Coreta-Gomes F., Moreno M.J., Loura L.M.S. (2019). Interaction of Bile Salts with Lipid Bilayers: An Atomistic Molecular Dynamics Study. Front. Physiol..

[26] Lomize A.L., Pogozheva I.D. (2019). Physics-Based Method for Modeling Passive Membrane Permeability and Translocation Pathways of Bioactive Molecules. J. Chem. Inf. Model..

[27] Cardenas A.E., Shrestha R., Webb L.J., Elber R. (2015). Membrane permeation of a peptide: It is better to be positive. J. Phys. Chem. B.

[28] Coreta-Gomes F.M., Martins P.A.T., Velazquez-Campoy A., Vaz W.L.C., Geraldes C.F.G., Moreno M.J. (2015). Interaction of Bile Salts with Model Membranes Mimicking the Gastrointestinal Epithelium: A Study by Isothermal Titration Calorimetry. Langmuir.

[29] Moreno M.J., Salvador A. (2023). Ligand’s Partition to the Lipid Bilayer Should Be Accounted for When Estimating Their Affinity to Proteins. Molecules.

[30] Moreno M.J., Filipe H.A.L., Cunha S.V.P., Ramos C.V., Martins P.A.T., Abel B., Loura L.M.S., Ambudkar S.V. (2023). Interaction of a Homologous Series of Amphiphiles with P-glycoprotein in a Membrane Environment-Contributions of Polar and Non-Polar Interactions. Pharmaceutics.

[31] Moreno M.J., Loura L.M.S., Martins J., Salvador A., Velazquez-Campoy A. (2022). Analysis of the Equilibrium Distribution of Ligands in Heterogeneous Media-Approaches and Pitfalls. Int. J. Mol. Sci..

[32] Heerklotz H., Keller S. (2013). How membrane partitioning modulates receptor activation: Parallel versus serial effects of hydrophobic ligands. Biophys. J..

[33] Seelig A., Li-Blatter X. (2023). P-glycoprotein (ABCB1)-weak dipolar interactions provide the key to understanding allocrite recognition, binding, and transport. Cancer Drug Resist..

[34] Pelkmans L., Helenius A. (2002). Endocytosis via caveolae. Traffic.

[35] Chai G.H., Xu Y., Chen S.Q., Cheng B., Hu F.Q., You J., Du Y.Z., Yuan H. (2016). Transport Mechanisms of Solid Lipid Nanoparticles across Caco-2 Cell Monolayers and their Related Cytotoxicology. ACS Appl. Mater. Interfaces.

[36] Neves A.R., Queiroz J.F., Costa Lima S.A., Figueiredo F., Fernandes R., Reis S. (2016). Cellular uptake and transcytosis of lipid-based nanoparticles across the intestinal barrier: Relevance for oral drug delivery. J. Colloid Interface Sci..

[37] Mäger I., Meyer A.H., Li J., Lenter M., Hildebrandt T., Leparc G., Wood M.J.A. (2017). Targeting blood-brain-barrier transcytosis-perspectives for drug delivery. Neuropharmacology.

[38] Thuenauer R., Müller S.K., Römer W. (2017). Pathways of protein and lipid receptor-mediated transcytosis in drug delivery. Expert Opin. Drug Deliv..

[39] Knyazev E., Nersisyan S., Tonevitsky A. (2021). Endocytosis and Transcytosis of SARS-CoV-2 across the Intestinal Epithelium and Other Tissue Barriers. Front. Immunol..

[40] Choi E.S., Shusta E.V. (2023). Strategies to identify, engineer, and validate antibodies targeting blood-brain barrier receptor-mediated transcytosis systems for CNS drug delivery. Expert Opin. Drug Deliv..

[41] Fortuna A., Alves G., Falcão A. (2007). The importance of permeability screening in drug discovery process: PAMPA, Caco-2 and rat everted gut assays. Curr. Top. Pharmacol..

[42] Matter H., Schmider W., Vogel H.G., Maas J., Hock F.J., Mayer D. (2013). In-Silico ADME Modeling. Drug Discovery and Evaluation: Safety and Pharmacokinetic Assays.

[43] Beresford A.P., Selick H.E., Tarbit M.H. (2002). The emerging importance of predictive ADME simulation in drug discovery. Drug Discov. Today.

[44] van de Waterbeemd H., Gifford E. (2003). ADMET in silico modelling: Towards prediction paradise?. Nat. Rev. Drug Discov..

[45] Lipinski C.A., Lombardo F., Dominy B.W., Feeney P.J. (1997). Experimental and computational approaches to estimate solubility and permeability in drug discovery and development settings. Adv. Drug Deliv. Rev..

[46] Kramer S.D., Aschmann H.E., Hatibovic M., Hermann K.F., Neuhaus C.S., Brunner C., Belli S. (2016). When barriers ignore the "rule-of-five". Adv. Drug Deliv. Rev..

[47] Kamenik A.S., Kraml J., Hofer F., Waibl F., Quoika P.K., Kahler U., Schauperl M., Liedl K.R. (2020). Macrocycle Cell Permeability Measured by Solvation Free Energies in Polar and Apolar Environments. J. Chem. Inf. Model..

[48] Doak B.C., Over B., Giordanetto F., Kihlberg J. (2014). Oral Druggable Space beyond the Rule of 5: Insights from Drugs and Clinical Candidates. Chem. Biol..

[49] Over B., Matsson P., Tyrchan C., Artursson P., Doak B.C., Foley M.A., Hilgendorf C., Johnston S.E., Lee M.D., Lewis R.J. (2016). Structural and conformational determinants of macrocycle cell permeability. Nat. Chem. Biol..

[50] Bockus A.T., Lexa K.W., Pye C.R., Kalgutkar A.S., Gardner J.W., Hund K.C.R., Hewitt W.M., Schwochert J.A., Glassey E., Price D.A. (2015). Probing the Physicochemical Boundaries of Cell Permeability and Oral Bioavailability in Lipophilic Macrocycles Inspired by Natural Products. J. Med. Chem..

[51] McKerrow J.H., Lipinski C.A. (2017). The rule of five should not impede anti-parasitic drug development. Int. J. Parasitol.-Drugs Drug Resist..

[52] Hermann K.F., Neuhaus C.S., Micallef V., Wagner B., Hatibovic M., Aschmann H.E., Paech F., Alvarez-Sanchez R., Kramer S.D., Belli S. (2017). Kinetics of lipid bilayer permeation of a series of ionisable drugs and their correlation with human transporter-independent intestinal permeability. Eur. J. Pharm. Sci..

[53] Muthas D., Boyer S., Hasselgren C. (2013). A critical assessment of modeling safety-related drug attrition. MedChemComm.

[54] Schneckener S., Grimbs S., Hey J., Menz S., Osmers M., Schaper S., Hillisch A., Goller A.H. (2019). Prediction of Oral Bioavailability in Rats: Transferring Insights from in Vitro Correlations to (Deep) Machine Learning Models Using in Silico Model Outputs and Chemical Structure Parameters. J. Chem. Inf. Model..

[55] Wu F.X., Zhou Y.Q., Li L.H., Shen X.H., Chen G.Y., Wang X.Q., Liang X.Y., Tan M.Y., Huang Z.N. (2020). Computational Approaches in Preclinical Studies on Drug Discovery and Development. Front. Chem..

[56] Volkova T.V., Perlovich G.L. (2023). Permeability of New Antifungal Fluconazole Derivatives through a Lipophilic Membrane: Experiment and Modeling. Molecules.

[57] Egan W.J., Merz K.M., Baldwin J.J. (2000). Prediction of drug absorption using multivariate statistics. J. Med. Chem..

[58] Moss G.P., Dearden J.C., Patel H., Cronin M.T.D. (2002). Quantitative structure-permeability relationships (QSPRs) for percutaneous absorption. Toxicol. Vitr..

[59] Digiesi V., Roque V.D., Vallaro M., Caron G., Ermondi G. (2021). Permeability prediction in the beyond-Rule-of 5 chemical space: Focus on cyclic hexapeptides. Eur. J. Pharm. Biopharm..

[60] Oja M., Maran U. (2015). Quantitative structure-permeability relationships at various pH values for acidic and basic drugs and drug-like compounds. SAR QSAR Environ. Res..

[61] Refsgaard H.H.F., Jensen B.F., Brockhoff P.B., Padkjær S.B., Guldbrandt M., Christensen M.S. (2005). In silico prediction of membrane permeability from calculated molecular parameters. J. Med. Chem..

[62] Filipe H.A.L., Salvador A., Silvestre J.M., Vaz W.L.C., Moreno M.J. (2014). Beyond Overton’s Rule: Quantitative Modeling of Passive Permeation through Tight Cell Monolayers. Mol. Phar..

[63] Zhang S., Thompson J.P., Xia J.C., Bogetti A.T., York F., Skillman A.G., Chong L.T., LeBard D.N. (2022). Mechanistic Insights into Passive Membrane Permeability of Drug-like Molecules from a Weighted Ensemble of Trajectories. J. Chem. Inf. Model..

[64] Rokitskaya T.I., Aleksandrova E.V., Korshunova G.A., Khailova L.S., Tashlitsky V.N., Luzhkov V.B., Antonenko Y.N. (2022). Membrane Permeability of Modified Butyltriphenylphosphonium Cations. J. Phys. Chem. B.

[65] Nagle J.F., Mathai J.C., Zeidel M.L., Tristram-Nagle S. (2008). Theory of passive permeability through lipid bilayers. J. Gen. Physiol..

[66] Oruç T., Küçük S.E., Sezer D. (2016). Lipid bilayer permeation of aliphatic amine and carboxylic acid drugs: Rates of insertion, translocation and dissociation from MD simulations. Phys. Chem. Chem. Phys..

[67] Dickson C.J., Hornak V., Pearlstein R.A., Duca J.S. (2017). Structure-Kinetic Relationships of Passive Membrane Permeation from Multiscale Modeling. J. Am. Chem. Soc..

[68] Coimbra J.T.S., Feghali R., Ribeiro R.P., Ramos M.J., Fernandes P.A. (2021). The importance of intramolecular hydrogen bonds on the translocation of the small drug piracetam through a lipid bilayer. RSC Adv..

[69] Swift R.V., Amaro R.E. (2013). Back to the Future: Can Physical Models of Passive Membrane Permeability Help Reduce Drug Candidate Attrition and Move Us Beyond QSPR?. Chem. Biol. Drug Des..

[70] Brocke S.A., Degen A., MacKerell A.D., Dutagaci B., Feig M. (2019). Prediction of Membrane Permeation of Drug Molecules by Combining an Implicit Membrane Model with Machine Learning. J. Chem. Inf. Model..

[71] Sun H.M. (2005). Predicting ADMET Properties by Projecting onto Chemical Space-Benefits and Pitfalls. Curr. Comput.-Aided Drug Des..

[72] Thummel K., Kunze K., Shen D. (1997). Enzyme-catalyzed processes of first-pass hepatic and intestinal drug extraction. Advanced Drug Delivery Reviews.

[73] Stuurman F., Nuijen B., Beijnen J., Schellens J. (2013). Oral Anticancer Drugs: Mechanisms of Low Bioavailability and Strategies for Improvement. Clinical Pharmacokinetics.

[74] Cabrera-Pérez M., Pham-The H. (2018). Computational modeling of human oral bioavailability: What will be next?. Expert Opin. Drug Discov..

[75] Yoshida F., Topliss J.G. (2000). QSAR model for drug human oral bioavailability. J. Med. Chem..

[76] Cabrera-Pérez M.A., Pham-The H., Bermejo M., Alvarez I.G., Alvarez M.G., Garrigues T.M. (2012). QSPR in Oral Bioavailability: Specificity or Integrality?. Mini-Rev. Med. Chem..

[77] Zhu J.Y., Wang J.M., Yu H.D., Li Y.Y., Hou T.J. (2011). Recent Developments of In Silico Predictions of Oral Bioavailability. Comb. Chem. High. Throughput Screen..

[78] Turner J.V., Glass B.D., Agatonovic-Kustrin S. (2003). Prediction of drug bioavailability based on molecular structure. Anal. Chim. Acta.

[79] Tian S., Li Y., Wang J., Zhang J., Hou T. (2011). ADME Evaluation in Drug Discovery. 9. Prediction of Oral Bioavailability in Humans Based on Molecular Properties and Structural Fingerprints. Molecular Pharmaceutics.

[80] Maeda K. (2023). Quantitative Prediction of Intestinal Absorption of Drugs from In Vitro Study: Utilization of Differentiated Intestinal Epithelial Cells Derived from Intestinal Stem Cells at Crypts. Drug Metab. Dispos..

[81] Garg R., Garg A. (2023). A Comprehensive Review on Recent Advances and Considerations for the Selection of Cell-based. Curr. Drug Deliv..

[82] Youhanna S., Lauschke V.M. (2021). The Past, Present and Future of Intestinal In Vitro Cell Systems for Drug Absorption Studies. J. Pharm. Sci..

[83] Michiba K., Maeda K., Kurimori K., Enomoto T., Shimomura O., Takeuchi T., Nishiyama H., Oda T., Kusuhara H. (2021). Characterization of the Human Intestinal Drug Transport with Ussing Chamber System Incorporating Freshly Isolated Human Jejunum. Drug Metab. Dispos..

[84] Lennernäs H., Nylander S., Ungell A.L. (1997). Jejunal permeability: A comparison between the ussing chamber technique and the single-pass perfusion in humans. Pharm. Res..

[85] Kansy M., Senner F., Gubernator K. (1998). Physicochemical high throughput screening: Parallel artificial membrane permeation assay in the description of passive absorption processes. J. Med. Chem..

[86] Di L., Kerns E.H., Fan K., McConnell O.J., Carter G.T. (2003). High throughput artificial membrane permeability assay for blood-brain barrier. Eur. J. Med. Chem..

[87] Diamond J.M., Katz Y. (1974). Interpretation of nonelectrolyte partition coefficients between dimyristoyl lecithin and water. J. Membr. Biol..

[88] Klopman G., Stefan L.R., Saiakhov R.D. (2002). ADME evaluation 2. A computer model for the prediction of intestinal absorption in humans. Eur. J. Pharm. Sci..

[89] Hou T., Wang J., Zhang W., Xu X. (2007). ADME evaluation in drug discovery. 6. Can oral bioavailability in humans be effectively predicted by simple molecular property-based rules?. J. Chem. Inf. Model..

[90] Eyer K., Paech F., Schuler F., Kuhn P., Kissner R., Belli S., Dittrich P.S., Krämer S.D. (2014). A liposomal fluorescence assay to study permeation kinetics of drug-like weak bases across the lipid bilayer. J. Control. Release.

[91] Gabba M., Frallicciardi J., van’t Klooster J., Henderson R., Syga Ł., Mans R., van Maris A.J.A., Poolman B. (2020). Weak Acid Permeation in Synthetic Lipid Vesicles and Across the Yeast Plasma Membrane. Biophys. J..

[92] Frallicciardi J., Melcr J., Siginou P., Marrink S.J., Poolman B. (2022). Membrane thickness, lipid phase and sterol type are determining factors in the permeability of membranes to small solutes. Nat. Commun..

[93] Sharifian Gh M. (2021). Recent Experimental Developments in Studying Passive Membrane Transport of Drug Molecules. Mol. Pharm..

[94] Schaich M., Cama J., Al Nahas K., Sobota D., Sleath H., Jahnke K., Deshpande S., Dekker C., Keyser U.F. (2019). An Integrated Microfluidic Platform for Quantifying Drug Permeation across Biomimetic Vesicle Membranes. Mol. Pharm..

[95] Sarmento B., Andrade F., da Silva S.B., Rodrigues F., das Neves J., Ferreira D. (2012). Cell-based in vitro models for predicting drug permeability. Expert Opin. Drug Metab. Toxicol..

[96] Hidalgo I.J., Raub T.J., Borchardt R.T. (1989). Characterization of the human colon carcinoma cell line (Caco-2) as a model system for intestinal epithelial permeability. Gastroenterology.

[97] Pinto M., Robineleon S., Appay M.D., Kedinger M., Triadou N., Dussaulx E., Lacroix B., Simonassmann P., Haffen K., Fogh J. (1983). Enterocyte-like differentiation and polarization of the human-colon carcinoma cell-line Caco-2 in culture. Biol. Cell.

[98] Balimane P.V., Chong S. (2005). Cell culture-based models for intestinal permeability: A critique. Drug Discov. Today.

[99] Volpe D.A. (2020). Advances in cell-based permeability assays to screen drugs for intestinal absorption. Expert Opin. Drug Discov..

[100] Gartzke D., Fricker G. (2014). Establishment of Optimized MDCK Cell Lines for Reliable Efflux Transport Studies. J. Pharm. Sci..

[101] Harwood M.D., Zettl K., Weinheimer M., Pilla-Reddy V., Shen H., Jacobs F., Chu X.Y., Huth F., Nakakariya M., Chothe P.P. (2023). Interlaboratory Variability in the Madin-Darby Canine Kidney Cell Proteome. Mol. Pharm..

[102] Hidalgo I.J. (2001). Assessing the absorption of new pharmaceuticals. Curr. Top. Med. Chem..

[103] Hämmerle S.P., Rothen-Rutishauser B., Krämer S.D., Günthert M., Wunderli-Allenspach H. (2000). P-glycoprotein in cell cultures: A combined approach to study expression, localisation, and functionality in the confocal microscope. Eur. J. Pharm. Sci..

[104] Pastan I., Gottesman M.M., Ueda K., Lovelace E., Rutherford A.V., Willingham M.C. (1988). A retrovirus carrying an MDR1 cDNA confers multidrug resistance and polarized expression of p-glycoprotein in MDCK cells. Proc. Natl. Acad. Sci. USA.

[105] Veszelka S., Tóth A., Walter F.R., Tóth A.E., Gróf I., Mészáros M., Bocsik A., Hellinger É., Vastag M., Rákhely G. (2018). Comparison of a Rat Primary Cell-Based Blood-Brain Barrier Model with Epithelial and Brain Endothelial Cell Lines: Gene Expression and Drug Transport. Front. Mol. Neurosci..

[106] Jin X., Thu-Lan L., Reese N., Gaona H., Collazo-Velez V., Chau V., Potter B., Sousa J.C., Olmeda R., Li Q. (2014). Comparison of MDCK-MDR1 and Caco-2 cell based permeability assays for anti-malarial drug screening and drug investigations. J. Pharmacol. Toxicol. Methods.

[107] Bicker J., Alves G., Fortuna A., Soares-da-Silva P., Falcão A. (2018). In vitro assessment of the interactions of dopamine β-hydroxylase inhibitors with human P-glycoprotein and Breast Cancer Resistance Protein. Eur. J. Pharm. Sci..

[108] Mease K., Sane R., Podila L., Taub M.E. (2012). Differential Selectivity of Efflux Transporter Inhibitors in Caco-2 and MDCK-MDR1 Monolayers: A Strategy to Assess the Interaction of a New Chemical Entity with P-gp, BCRP, and MRP2. J. Pharm. Sci..

[109] Tang F.X., Horie K., Borchardt R.T. (2002). Are MDCK cells transfected with the Human MDR1 gene a good model of the human intestinal mucosa?. Pharm. Res..

[110] Putnam W.S., Ramanathan S., Pan L., Takahashi L.H., Benet L.Z. (2002). Functional characterization of monocarboxylic acid, large neutral amino acid, bile acid and peptide transporters, and P-glycoprotein in MDCK and Caco-2 cells. J. Pharm. Sci..

[111] Chu X.Y., Bleasby K., Evers R. (2013). Species differences in drug transporters and implications for translating preclinical findings to humans. Expert Opin. Drug Metab. Toxicol..

[112] Hellinger E., Veszelka S., Toth A.E., Walter F., Kittel A., Bakk M.L., Tihanyi K., Hada V., Nakagawa S., Thuy D.H.D. (2012). Comparison of brain capillary endothelial cell-based and epithelial (MDCK-MDR1, Caco-2, and VB-Caco-2) cell-based surrogate blood-brain barrier penetration models. Eur. J. Pharm. Biopharm..

[113] Tokuda S., Furuse M. (2015). Claudin-2 Knockout by TALEN-Mediated Gene Targeting in MDCK Cells: Claudin-2 Independently Determines the Leaky Property of Tight Junctions in MDCK Cells. PLoS ONE.

[114] Praça C., Rosa S., Sevin E., Cecchelli R., Dehouck M., Ferreira L. (2019). Derivation of Brain Capillary-like Endothelial Cells from Human Pluripotent Stem Cell-Derived Endothelial Progenitor Cells. Stem Cell Rep..

[115] Geldenhuys W., Mohammad A., Adkins C., Lockman P. (2015). Molecular determinants of blood-brain barrier permeation. Ther. Deliv..

[116] Bicker J., Alves G., Fortuna A., Falcão A. (2014). Blood–brain barrier models and their relevance for a successful development of CNS drug delivery systems: A review. Eur. J. Pharm. Biopharm..

[117] Nakagawa S., Deli M., Kawaguchi H., Shimizudani T., Shimono T., Kittel A., Tanaka K., Niwa M. (2009). A new blood-brain barrier model using primary rat brain endothelial cells, pericytes and astrocytes. Neurochem. Int..

[118] VanDussen K., Marinshaw J., Shaikh N., Miyoshi H., Moon C., Tarr P., Ciorba M., Stappenbeck T. (2015). Development of an enhanced human gastrointestinal epithelial culture system to facilitate patient-based assays. Gut.

[119] vandeWaterbeemd H., Camenisch G., Folkers G., Raevsky O.A. (1996). Estimation of Caco-2 cell permeability using calculated molecular descriptors. Quant. Struct.-Act. Relatsh..

[120] Sherer E.C., Verras A., Madeira M., Hagmann W.K., Sheridan R.P., Roberts D., Bleasby K., Cornell W.D. (2012). QSAR Prediction of Passive Permeability in the LLC-PK1 Cell Line: Trends in Molecular Properties and Cross-Prediction of Caco-2 Permeabilities. Mol. Inform..

[121] Singh K.P., Gupta S., Basant N. (2015). In silico prediction of cellular permeability of diverse chemicals using qualitative and quantitative SAR modeling approaches. Chemom. Intell. Lab. Syst..

[122] Wang N.N., Dong J., Deng Y.H., Zhu M.F., Wen M., Yao Z.J., Lu A.P., Wang J.B., Cao D.S. (2016). ADME Properties Evaluation in Drug Discovery: Prediction of Caco-2 Cell Permeability Using a Combination of NSGA-II and Boosting. J. Chem. Inf. Model..

[123] Wang Y.K., Chen X.B. (2020). QSPR model for Caco-2 cell permeability prediction using a combination of HQPSO and dual-RBF neural network. RSC Adv..

[124] Artursson P., Palm K., Luthman K. (1996). Caco-2 monolayers in experimental and theoretical predictions of drug transport. Adv. Drug Deliv. Rev..

[125] Press B., Di Grandi D. (2008). Permeability for Intestinal Absorption: Caco-2 Assay and Related Issues. Curr. Drug Metab..

[126] Volpe D.A. (2008). Variability in Caco-2 and MDCK cell-based intestinal permeability assays. J. Pharm. Sci..

[127] Subramanian G., Kitchen D.B. (2006). Computational approaches for modeling human intestinal absorption and permeability. J. Mol. Modell..

[128] Clark R.D., Daga P.R. (2019). Building a Quantitative Structure-Property Relationship (QSPR) Model. Bioinform. Drug Discov..

[129] Lomize A.L., Hage J.M., Schnitzer K., Golobokov K., LaFaive M.B., Forsyth A.C., Pogozheva I.D. (2019). PerMM: A Web Tool and Database for Analysis of Passive Membrane Permeability and Translocation Pathways of Bioactive Molecules. J. Chem. Inf. Model..

[130] Juracka J., Srejber M., Melikova M., Bazgier V., Berka K. (2019). MolMeDB: Molecules on Membranes Database. Database-J. Biol. Databases Curation.

[131] Avdeef A. (2012). Permeability: Caco-2/MDCK.

[132] Irvine J.D., Takahashi L., Lockhart K., Cheong J., Tolan J.W., Selick H.E., Grove J.R. (1999). MDCK (Madin-Darby canine kidney) cells: A tool for membrane permeability screening. J. Pharm. Sci..

[133] Yazdanian M., Glynn S.L., Wright J.L., Hawi A. (1998). Correlating partitioning and Caco-2 cell permeability of structurally diverse small molecular weight compounds. Pharm. Res..

[134] Artursson P., Karlsson J. (1991). Correlation between Oral-Drug Absorption in Humans and Apparent Drug Permeability Coefficients in Human Intestinal Epithelial (Caco-2) Cells. Biochem. Biophys. Res. Commun..

[135] Chong S.H., Dando S.A., Soucek K.M., Morrison R.A. (1996). In vitro permeability through Caco-2 cells is not quantitatively predictive of in vivo absorption for peptide-like drugs absorbed via the dipeptide transporter system. Pharm. Res..

[136] Pade V., Stavchansky S. (1998). Link between drug absorption solubility and permeability measurements in Caco-2 cells. J. Pharm. Sci..

[137] Lee J.B., Zgair A., Taha D.A., Zang X.W., Kagan L., Kim T.H., Kim M.G., Yun H.Y., Fischer P.M., Gershkovich P. (2017). Quantitative analysis of lab-to-lab variability in Caco-2 permeability assays. Eur. J. Pharm. Biopharm..

[138] Gres M.C., Julian B., Bourrie M., Meunier V., Roques C., Berger M., Boulenc X., Berger Y., Fabre G. (1998). Correlation between oral drug absorption in humans, and apparent drug permeability in TC-7 cells, a human epithelial intestinal cell line: Comparison with the parental Caco-2 cell line. Pharm. Res..

[139] Jung S.J., Choi S.O., Um S.Y., Kim J.I., Choo H.Y.P., Choi S.Y., Chung S.Y. (2006). Prediction of the permeability of drugs through study on quantitative structure-permeability relationship. J. Pharm. Biomed. Anal..

[140] Kerns E.H., Di L., Petusky S., Farris M., Ley R., Jupp P. (2004). Combined application of parallel artificial membrane permeability assay and Caco-2 permeability assays in drug discovery. J. Pharm. Sci..

[141] Larregieu C.A., Benet L.Z. (2013). Drug Discovery and Regulatory Considerations for Improving In Silico and In Vitro Predictions that Use Caco-2 as a Surrogate for Human Intestinal Permeability Measurements. AAPS J..

[142] Sambuy Y., De Angelis I., Ranaldi G., Scarino M.L., Stammati A., Zucco F. (2005). The Caco-2 cell line as a model of the intestinal barrier: Influence of cell and culture-related factors on Caco-2 cell functional characteristics. Cell Biol. Toxicol..

[143] Oltra-Noguera D., Mangas-Sanjuan V., Centelles-Sangüesa A., Gonzalez-Garcia I., Sanchez-Castaño G., Gonzalez-Alvarez M., Casabo V.-G., Merino V., Gonzalez-Alvarez I., Bermejo M. (2015). Variability of permeability estimation from different protocols of subculture and transport experiments in cell monolayers. J. Pharmacol. Toxicol. Methods.

[144] Delie F., Rubas W. (1997). A human colonic cell line sharing similarities with enterocytes as a model to examine oral absorption: Advantages and limitations of the Caco-2 model. Crit. Rev. Ther. Drug Carr. Syst..

[145] Hayeshi R., Hilgendorf C., Artursson P., Augustijns P., Brodin B., Dehertogh P., Fisher K., Fossati L., Hovenkamp E., Korjamo T. (2008). Comparison of drug transporter gene expression and functionality in Caco-2 cells from 10 different laboratories. Eur. J. Pharm. Sci..

[146] Walter E., Kissel T. (1995). Heterogeneity in the human intestinal cell line Caco-2 leads to differences in transepithelial transport. Eur. J. Pharm. Sci..

[147] Behrens I., Kamm W., Dantzig A.H., Kissel T. (2004). Variation of peptide transporter (PepT1 expression in Caco-2 cells as a function and HPT1) of cell origin. J. Pharm. Sci..

[148] D’Souza V.M., Shertzer H.G., Menon A.G., Pauletti G.M. (2003). High glucose concentration in isotonic media alters Caco-2 cell permeability. AAPS Pharmsci.

[149] DeMarco V.G., Li N., Thomas J., West C.M., Neu J. (2003). Glutamine and barrier function in cultured Caco-2 epithelial cell monolayers. J. Nutr..

[150] Ranaldi G., Consalvo R., Sambuy Y., Scarino M.L. (2003). Permeability characteristics of parental and clonal human intestinal Caco-2 cell lines differentiated in serum-supplemented and serum-free media. Toxicol. Vitr..

[151] Yu H.S., Cook T.J., Sinko P.J. (1997). Evidence for diminished functional expression of intestinal transporters in Caco-2 cell monolayers at high passages. Pharm. Res..

[152] Lu S., Gough A.W., Bobrowski W.F., Stewart B.H. (1996). Transport properties are not altered across Caco-2 cells with heightened TEER despite underlying physiological and ultrastructural changes. J. Pharm. Sci..

[153] Behrens I., Kissel T. (2003). Do cell culture conditions influence the carrier-mediated transport of peptides in Caco-2 cell monolayers?. Eur. J. Pharm. Sci..

[154] Hosoya K., Kim K.J., Lee V.H.L. (1996). Age-dependent expression of P-glycoprotein gp170 in Caco-2 cell monolayers. Pharm. Res..

[155] Markowska M., Oberle R., Juzwin S., Hsu C.P., Gryszkiewicz M., Streeter A.J. (2001). Optimizing Caco-2 cell monolayers to increase throughput in drug intestinal absorption analysis. J. Pharmacol. Toxicol. Methods.

[156] Vachon P.H., Beaulieu J.F. (1992). Transient mosaic patterns of morphological and functional-differentiation in the Caco-2 cell-line. Gastroenterology.

[157] Herold G., Rogler D., Rogler G., Stange E.F. (1994). Morphology of Caco-2 cells varies in different cell batches. Vitr. Cell. Dev. Biol.-Anim..

[158] Horie K., Tang F.X., Borchardt R.T. (2003). Isolation and characterization of Caco-2 subclones expressing high levels of multidrug resistance protein efflux transporter. Pharm. Res..

[159] Turco L., Catone T., Caloni F., Di Consiglio E., Testai E., Stammati A. (2011). Caco-2/TC7 cell line characterization for intestinal absorption: How reliable is this in vitro model for the prediction of the oral dose fraction absorbed in human?. Toxicol. Vitr..

[160] Spring K.R. (1998). Routes and mechanism of fluid transport by epithelia. Annu. Rev. Physiol..

[161] Angelis I.D., Turco L. (2011). Caco-2 cells as a model for intestinal absorption. Curr. Protoc. Toxicol..

[162] Santos A., Rodrigues A.M., Sobral A.J.F.N., Monsanto P.V., Vaz W.L.C., Moreno M.J. (2009). Early Events in Photodynamic Therapy: Chemical and Physical Changes in a POPC:Cholesterol Bilayer due to Hematoporphyrin IX-mediated Photosensitization. Photochem. Photobiol..

[163] Jovanovic O., Skulj S., Pohl E.E., Vazdar M. (2019). Covalent modification of phosphatidylethanolamine by 4-hydroxy-2-nonenal increases sodium permeability across phospholipid bilayer membranes. Free Radic. Biol. Med..

[164] Meyer T.N., Schwesinger C., Ye J.M., Denker B.M., Nigam S.K. (2001). Reassembly of the tight junction after oxidative stress depends on tyrosine kinase activity. J. Biol. Chem..

[165] Dubois N., Muñoz-Garcia J., Heymann D., Renodon-Cornière A. (2023). High glucose exposure drives intestinal barrier dysfunction by altering its morphological, structural and functional properties. Biochem. Pharmacol..

[166] Morresi C., Cianfruglia L., Sartini D., Cecati M., Fumarola S., Emanuelli M., Armeni T., Ferretti G., Bacchetti T. (2019). Effect of High Glucose-Induced Oxidative Stress on Paraoxonase 2 Expression and Activity in Caco-2 Cells. Cells.

[167] Briske-Anderson M.J., Finley J.W., Newman S.M. (1997). The influence of culture time and passage number on the morphological and physiological development of Caco-2 cells. Proc. Soc. Exp. Biol. Med..

[168] Hubatsch I., Ragnarsson E.G.E., Artursson P. (2007). Determination of drug permeability and prediction of drug absorption in Caco-2 monolayers. Nat. Protoc..

[169] Moreira L.N., Feltrin C., Gonçalves J.E., de Castro W.V., Simoes C.M.O., de Pádua R.M., Cortes S.F., Braga F.C. (2020). Determination of L-(+)-bornesitol, the hypotensive constituent of Hancornia speciosa, in rat plasma by LC-MS/MS and its application on a pharmacokinetic study. Biomed. Pharmacother..

[170] Rodrigues E.T., Nascimento S.F., Pires C.L., Godinho L.P., Churro C., Moreno M.J., Pardal M.A. (2021). Determination of intestinal absorption of the paralytic shellfish toxin GTX-5 using the Caco-2 human cell model. Environ. Sci. Pollut. Res..

[171] Artursson P. (1990). Epithelial transport of drugs in cell-culture.1. A model for studying the passive diffusion of drugs over intestinal absorptive (Caco-2) cells. J. Pharm. Sci..

[172] Lechanteur A., Almeida A., Sarmento B. (2017). Elucidation of the impact of cell culture conditions of Caco-2 cell monolayer on barrier integrity and intestinal permeability. Eur. J. Pharm. Biopharm..

[173] DiMarco R.L., Hunt D.R., Dewi R.E., Heilshorn S.C. (2017). Improvement of paracellular transport in the Caco-2 drug screening model using protein-engineered substrates. Biomaterials.

[174] Yamashita S., Furubayashi T., Kataoka M., Sakane T., Sezaki H., Tokuda H. (2000). Optimized conditions for prediction of intestinal drug permeability using Caco-2 cells. Eur. J. Pharm. Sci..

[175] Saha P., Kou J.H. (2000). Effect of solubilizing excipients on permeation of poorly water-soluble compounds across Caco-2 cell monolayers. Eur. J. Pharm. Biopharm..

[176] Aungst B.J., Nguyen N.H., Bulgarelli J.P., Oates-Lenz K. (2000). The influence of donor and reservoir additives on Caco-2 permeability and secretory transport of HIV protease inhibitors and other lipophilic compounds. Pharm. Res..

[177] Neuhoff S., Artursson P., Zamora I., Ungell A.L. (2006). Impact of extracellular protein binding on passive and active drug transport across Caco-2 cells. Pharm. Res..

[178] Krishna G., Chen K.-J., Lin C.-C., Nomeir A.A. (2001). Permeability of lipophilic compounds in drug discovery using in-vitro human absorption model, Caco-2. Int. J. Pharm..

[179] Katneni K., Charman S.A., Porter C.J.H. (2008). Use of plasma proteins as solubilizing agents in in vitro permeability experiments: Correction for unbound drug concentration using the reciprocal permeability approach. J. Pharm. Sci..

[180] Yu H.L., Huang Q.R. (2011). Investigation of the Absorption Mechanism of Solubilized Curcumin Using Caco-2 Cell Monolayers. J. Agric. Food Chem..

[181] Neuhoff S., Ungell A.-L., Zamora I., Artursson P. (2003). pH-Dependent Bidirectional Transport of Weakly Basic Drugs Across Caco-2 Monolayers: Implications for Drug–Drug Interactions. Pharm. Res..

[182] Pires C.L., Silva I.M.V., Coimbra M.A., Moreno M.J., Coreta-Gomes F. (2022). Effect of Coffee on the Bioavailability of Sterols. Foods.

[183] Broeders J.J.W., van Eijkeren J.C.H., Blaauboer B.J., Hermens J.L.M. (2012). Transport of Chlorpromazine in the Caco-2 Cell Permeability Assay: A Kinetic Study. Chem. Res. Toxicol..

[184] Wang Y., Cao J., Wang X., Zeng S. (2010). Stereoselective Transport and Uptake of Propranolol Across Human Intestinal Caco-2 Cell Monolayers. Chirality.

[185] Riede J., Umehara K.-I., Schweigler P., Huth F., Schiller H., Camenisch G., Poller B. (2019). Examining P-gp efflux kinetics guided by the BDDCS-Rational selection of in vitro assay designs and mathematical models. Eur. J. Pharm. Sci..

[186] Kishimoto W., Ishiguro N., Ludwig-Schwellinger E., Ebner T., Schaefer O. (2014). In Vitro Predictability of Drug-Drug Interaction Likelihood of P-Glycoprotein-Mediated Efflux of Dabigatran Etexilate Based on [I]2IC50 Threshold. Drug Metab. Dispos..

[187] Karlsson J., Artursson P. (1991). A method for the determination of cellular permeability coefficients and aqueous boundary layer thickness in monolayers of intestinal epithelial (Caco-2) cells grown in permeable filter chambers. Int. J. Pharm..

[188] Korjamo T., Heikkinen A.T., Waltari P., Mönkkönen J. (2008). The Asymmetry of the Unstirred Water Layer in Permeability Experiments. Pharm. Res..

[189] Bunchongprasert K., Shao J. (2020). Impact of Media in Transport Study on Cell Monolayer Integrity and Permeability. J. Pharm. Sci..

[190] Zeng Z., Shen Z.L., Zhai S., Xu J.L., Liang H., Shen Q., Li Q.Y. (2017). Transport of curcumin derivatives in Caco-2 cell monolayers. Eur. J. Pharm. Biopharm..

[191] Almeida A.S., Silva B., Remião F., Fernandes C. (2023). Assessment of the Permeability of 3,4-Methylenedioxypyrovalerone (MDPV) across the Caco-2 Monolayer for Estimation of Intestinal Absorption and Enantioselectivity. Int. J. Mol. Sci..

[192] Jarc T., Novak M., Hevir N., Rižner T.L., Kreft M.E., Kristan K. (2019). Demonstrating suitability of the Caco-2 cell model for BCS-based biowaiver according to the recent FDA and ICH harmonised guidelines. J. Pharm. Pharmacol..

[193] Zhang L., Zheng Y., Chow M.S., Zuo Z. (2004). Investigation of intestinal absorption and disposition of green tea catechins by Caco-2 monolayer model. Int. J. Pharm..

[194] Buyukozturk F., Benneyan J.C., Carrier R.L. (2010). Impact of emulsion-based drug delivery systems on intestinal permeability and drug release kinetics. J. Control. Release.

[195] Zucco F., Batto A.F., Bises G., Chambaz J., Chiusolo A., Consalvo R., Cross H., Dal Negro G., de Angelis I., Fabre G. (2005). An inter-laboratory study to evaluate the effects of medium composition on the differentiation and barrier function of Caco-2 cell lines. Atla-Altern. Lab. Anim..

[196] Wilson G., Hassan I.F., Dix C.J., Williamson I., Shah R., Mackay M., Artursson P. (1990). Transport and permeability properties of human Caco-2 cells—An in vitro model of the intestinal epithelial cell barrier. J. Control. Release.

[197] Hiebl V., Schachner D., Ladurner A., Heiss E.H., Stangl H., Dirsch V.M. (2020). Caco-2 Cells for Measuring Intestinal Cholesterol Transport—Possibilities and Limitations. Biol. Proced. Online.

[198] Ingels F., Deferme S., Destexhe E., Oth M., Van den Mooter G., Augustijns P. (2002). Simulated intestinal fluid as transport medium in the Caco-2 cell culture model. Int. J. Pharm..

[199] Buckley S.T., Fischer S.M., Fricker G., Brandl M. (2012). In vitro models to evaluate the permeability of poorly soluble drug entities: Challenges and perspectives. Eur. J. Pharm. Sci..

[200] Gomes F.M.C., Geraldes C.F.G., Vaz W.L.C., Moreno M.J. (2010). Emulsification of Cholesterol in Bile Salt Micelles: Relevance for Cholesterol Absorption. Proc. Biophys. J..

[201] Moreno M.J., Bastos M., Velazquez-Campoy A. (2010). Partition of amphiphilic molecules to lipid bilayers by isothermal titration calorimetry. Anal. Biochem..

[202] Delamaza A., Parra J.L. (1995). Vesicle-micelle structural transitions of phospholipid-bilayers and sodium dodecyl-sulfate. Langmuir.

[203] Bogman K., Erne-Brand F., Alsenz J., Drewe J. (2003). The role of surfactants in the reversal of active transport mediated by multidrug resistance proteins. J. Pharm. Sci..

[204] Ollila F., Slotte J.P. (2001). A thermodynamic study of bile salt interactions with phosphatidylcholine and sphingomyelin unilamellar vesicles. Langmuir.

[205] Schuldes H., Dolderer J.H., Zimmer G., Knobloch J., Bickeboller R., Jonas D., Woodcock B.G. (2001). Reversal of multidrug resistance and increase in plasma membrane fluidity in CHO cells with R-verapamil and bile salts. Eur. J. Cancer.

[206] Tan A.M., Ziegler A., Steinbauer B., Seelig J. (2002). Thermodynamics of sodium dodecyl sulfate partitioning into lipid membranes. Biophys. J..

[207] Laitinen L., Takala E., Vuorela H., Vuorela P., Kaukonen A.M., Marvola M. (2007). Anthranoid laxatives influence the absorption of poorly permeable drugs in human intestinal cell culture model (Caco-2). Eur. J. Pharm. Biopharm..

[208] Da Violante G., Zerrouk N., Richard I., Provot G., Chaumeil J.C., Arnaud P. (2002). Evaluation of the cytotoxicity effect of dimethyl sulfoxide (DMSO) on Caco_2_/TC7 colon tumor cell cultures. Biol. Pharm. Bull..

[209] Bohnert T., Gan L.S. (2013). Plasma Protein Binding: From Discovery to Development. J. Pharm. Sci..

[210] Rafols C., Amezqueta S., Fuguet E., Bosch E. (2018). Molecular interactions between warfarin and human (HSA) or bovine (BSA) serum albumin evaluated by isothermal titration calorimetry (ITC), fluorescence spectrometry (FS) and frontal analysis capillary electrophoresis (FA/CE). J. Pharm. Biomed. Anal..

[211] Zhuo W.L., Peng X.L., Lin X. (2018). Insights into the interaction mechanism between tiagabine hydrochloride and two serum albumins. RSC Adv..

[212] Pantusa M., Sportelli L., Bartucci R. (2008). Spectroscopic and calorimetric studies on the interaction of human serum albumin with DPPC/PEG:2000-DPPE membranes. Eur. Biophys. J. Biophys. Lett..

[213] Reddy A.C.P., Sudharshan E., Rao A.G.A., Lokesh B.R. (1999). Interaction of curcumin with human serum albumin—A spectroscopic study. Lipids.

[214] Thomae A.V., Wunderli-Allenspach H., Kramer S.D. (2005). Permeation of aromatic carboxylic acids across lipid bilayers: The pH-partition hypothesis revisited. Biophys. J..

[215] Bretschneider B., Brandsch M., Neubert R. (1999). Intestinal transport of beta-lactam antibiotics: Analysis of the affinity at the H+/peptide symporter (PEPT1), the uptake into Caco-2 cell monolayers and the transepithelial flux. Pharm. Res..

[216] Ieiri I., Doi Y., Maeda K., Sasaki T., Kimura M., Hirota T., Chiyoda T., Miyagawa M., Irie S., Iwasaki K. (2012). Microdosing Clinical Study: Pharmacokinetic, Pharmacogenomic (SLCO2B1), and Interaction (Grapefruit Juice) Profiles of Celiprolol Following the Oral Microdose and Therapeutic Dose. J. Clin. Pharmacol..

[217] Petri N., Tannergren C., Rungstad D., Lennernäs H. (2004). Transport characteristics of fexofenadine in the Caco-2 cell model. Pharm. Res..

[218] Li Y., Shin Y., Yu C., Kosmeder J., Hirschelman W., Pezzuto J., van Breemen R. (2003). Increasing the throughput and productivity of Caco-2 cell permeability assays using liquid chromatography-mass spectrometry: Application to resveratrol absorption and metabolism. Comb. Chem. High Throughput Screen..

[219] Laitinen L., Kangas H., Kaukonen A.M., Hakala K., Kotiaho T., Kostiainen R., Hirvonen J. (2003). N-in-one permeability studies of heterogeneous sets of compounds across Caco-2 cell monolayers. Pharm. Res..

[220] Balasubramanian S., Lynch J., Barry P. (1997). Concentration dependence of sodium permeation and sodium ion interactions in the cyclic AMP-gated channels of mammalian olfactory receptor neurons. J. Membr. Biol..

[221] Yang L., Feng F., Fawcett J.P., Tucker I.G. (2015). Kinetic and equilibrium studies of bile salt-liposome interactions. J. Liposome Res..

[222] Bourgaux C., Couvreur P. (2014). Interactions of anticancer drugs with biomembranes: What can we learn from model membranes?. J. Control. Release.

[223] Lombardi D., Cuenoud B., Krämer S. (2009). Lipid membrane interactions of indacaterol and salmeterol: Do they influence their pharmacological properties?. Eur. J. Pharm. Sci..

[224] Tse C., Comer J., Wang Y., Chipot C. (2018). Link between Membrane Composition and Permeability to Drugs. J. Chem. Theory Comput..

[225] Zocher F., van der Spoel D., Pohl P., Hub J. (2013). Local Partition Coefficients Govern Solute Permeability of Cholesterol-Containing Membranes. Biophys. J..

[226] Moreno M.J., Estronca L., Vaz W.L.C. (2006). Translocation of phospholipids and dithionite permeability in liquid-ordered and liquid-disordered membranes. Biophys. J..

[227] Saito H., Shinoda W. (2011). Cholesterol Effect on Water Permeability through DPPC and PSM Lipid Bilayers: A Molecular Dynamics Study. J. Phys. Chem. B.

[228] Lange Y., Tabei S.M.A., Ye J., Steck T.L. (2013). Stability and Stoichiometry of Bilayer Phospholipid-Cholesterol Complexes: Relationship to Cellular Sterol Distribution and Homeostasis. Biochemistry.

[229] Konsoula R., Barile F.A. (2005). Correlation of in vitro cytotoxicity with paracellular permeability in Caco-2 cells. Toxicol. Vitr..

[230] Lennernäs H. (1998). Human intestinal permeability. J. Pharm. Sci..

[231] Pires C.L., Praca C., Martins P.A.T., de Carvalho A., Ferreira L., Marques M.P.M., Moreno M.J. (2021). Re-Use of Caco-2 Monolayers in Permeability Assays-Validation Regarding Cell Monolayer Integrity. Pharmaceutics.

[232] Paketuryte V., Petrauskas V., Zubriene A., Abian O., Bastos M., Chen W.-Y., Moreno M.J., Krainer G., Linkuviene V., Sedivy A. (2021). Uncertainty in protein-ligand binding constants: Asymmetric confidence intervals versus standard errors. Eur. Biophys. J. Biophys. Lett..

[233] Natoli M., Leoni B.D., D’Agnano I., Zucco F., Felsani A. (2012). Good Caco-2 cell culture practices. Toxicol. Vitr..

[234] Gao J., Hugger E.D., Beck-Westermeyer M.S., Borchardt R.T. (2001). Estimating intestinal mucosal permeation of compounds using Caco-2 cell monolayers. Curr. Protoc. Pharmacol..

[235] Paixao P., Gouveia L.F., Morais J.A.G. (2010). Prediction of the in vitro permeability determined in Caco-2 cells by using artificial neural networks. Eur. J. Pharm. Sci..

[236] Pires C.L., Rosa S., de Carvalho A.L.B., Ferreira L., Marques M.P.M., Moreno M.J. (2024). Re-use of Caco-2 monolayers for a higher throughput assessment of drug permeability–methodology and validation for passive permeation. Preprints.

[237] Thomae A.V., Koch T., Panse C., Wunderli-Allenspach H., Krämer S.D. (2007). Comparing the lipid membrane affinity and permeation of drug-like acids: The intriguing effects of cholesterol and charged lipids. Pharm. Res..

