# Supplementary material for: Improving the Accuracy of Permeability Data to Gain Predictive Power: Assessing Sources of Variability in Assays Using Cell Monolayers
Source: Membranes (Basel). 2024 Jul 14;14(7):157. doi: 10.3390/membranes14070157 (PMC11278619; doi:10.3390/membranes14070157)
Supplement: Supplementary file 1 [file membranes-14-00157-s001.zip › membranes-3067932-supplementary.pdf]

Supplementary material for :

Improving the Accuracy of Permeability Data to Gain Predictive Power: Assessing Sources of Variability in Assays using Cell Monolayers

Cristiana L. Pires [1,2,*] and Maria João Moreno [1,2,*]

[1] Coimbra Chemistry Center - Institute of Molecular Sciences (CQC-IMS), University of Coimbra, 3004-535 Coimbra, Portugal
[2] Chemistry Department, Faculty of Science and Technology, University of Coimbra, 3004-535 Coimbra, Portugal
* Correspondence: mmoreno@ci.uc.pt and cristiana.lages.pires@gmail.com; Tel.: +351-239854481

Summary of the analysis of the impact of distinct variability sources

Table S1. Summary of the usual effect on the observed P_{app} for several sources of variability related to the cell culture protocols (pre- and post-seeding in the Transwell™ inserts).

Variability sources	Effect
**Cell supplier**[a]	Higher heterogeneity of cells provided from ATCC as compared to cells from DKFK, with respect to morphology and PEpT1 distribution [1,2]
**Culture media composition**	Higher glucose concentrations (25mM) increased the oxidative stress in cells. This affected mostly the passive permeability leading to an increase of P_{app} [3]
	Serum or glutamine depletion affected mostly the maturation of tight junctions during the differentiation period, which led to an increase of the paracellular permeability [4,5]
	The presence of the peptone substrate of PepT1 transporter increased its expression levels in the cells [6]
**Passage number**	Low passage numbers resulted in less tight cell monolayers compared to high passage numbers [7]
**Seeding density**	High passages numbers together with a high seeding density may lead to the formation of multiple cell layers [6,8]<br>Maximum level of P-gp was obtained with intermediate seeding density [6]
**Day post-seeding**	Affected mainly the monolayer differentiation, with an increase in cell tightness [6] and P-gp and PepT1 expression levels [9]

**Material and coating of the membrane insert**	PET and PE membranes originated flat poorly differentiated monolayers, whether PC membranes originated columnar-shaped cells and fully differentiated monolayers [6].<br>PET membranes displayed the lowest and PC the highest paracellular permeability [1,6]. This difference may arise from variations in how the cell´s basolateral surface interacts with the porous material.<br>Collagen coating of the membrane increased the expression of PepT1 and P-gp [6]
**Diameter of the membrane insert (plate type)**	An increase of the membrane diameter led to a decrease in P_{app}, most significant for fast permeating compounds [10]
**Pore size of the membrane insert**	A pore size of 1 μm led to the formation of a single monolayer of cells, while for a pore size of 3 μm, the cells migrated to the opposite side of the membrane and formed a double cell layer [11]

[a] ATCC: American Type Culture Collection; DKFZ: German Cancer Research Center

Table S2. Summary of the usual effect on the observed P_{app} for several sources of variability related to the permeability assays protocols.

Variability sources	Effect on the observed P_{app}
**Addition of surfactants or co-solvents in the donor side**	In the case of a very lipophilic surfactants or co-solvents that interact with the cell monolayer, the overall result on P_{app} is the balance of distinct effects:<br>▪ Decrease cell monolayer integrity due to cytotoxicity and/or disruption of tight junctions, which lead to an increase in P_{app} for paracellular permeation [12]<br>▪ Change the membrane fluidity, leading to variations in P_{app} for transcellular passive permeation, usually the fluidity increases with an increase in P_{app}<br>▪ Modulate the activity of membrane proteins, either directly or through changes in the properties of the lipid bilayer. Most surfactants are modulators of efflux transporters, and depending on their concentration may behave as substrates or inhibitors. The overall effect is therefore case-specific, although it usually leads to inhibition of efflux of the test compound with an increase of P_{app} in A→B direction and a decreased of P_{app} in B→A direction<br><br>If the additive does not perturb the cell monolayer but interacts with the test compound, three outcomes may be observed:<br>▪ For compounds that were well solubilized in the transport medium, interaction with surfactant micelles decreases the fraction of compound available to interact with the cell monolayer, leading to a decreased P_{app} [12]<br>▪ For very hydrophobic compounds that formed large aggregates in the transport medium, the increase in solubility will increase the compound availability to interact with the cell monolayer, leading to an increase in P_{app} [13]<br>▪ In general, the presence of surfactants or co-solvents decreases the compound adsorption to apparatus, leading to an increase in the amount of compound available to interact with the cell monolayer, and an increase in the recovery and in P_{app} [13]<br><br>At high concentrations of co-solvents, the properties of the transport medium may be changed with effects on P_{app} even for well soluble compounds. An example is the case of a decrease in viscosity which leads to a decrease in P_{app} [12]
**Addition of serum albumin in the donor and/or acceptor side**	The addition of serum albumin in the transport medium leads to distinct effects on P_{app} depending on the compartment(s) where it is added:<br>▪ In the donor compartment only, has the same effects identified above for surfactants and co-solvents that do not interact with the cell monolayer, decreasing or increasing P_{app} depending on the hydrophobicity of the test compound [14,15]<br>▪ In the acceptor compartment only, decreases the amount of compound adsorbed to the apparatus and retained by the cell monolayer, leading to an increase in recovery and in P_{app} of very lipophilic compounds [16]<br>▪ When added to both compartments, the effects are a combination of those described above. An increase in compound recovery is always observed, but the effect on P_{app} depends on the properties of the test compound [17]

**pH (apical/basolateral)**	The pH of the transport medium in the donor compartment influences the fraction of neutral compound and therefore its permeability through the cell monolayer. For compounds that permeate passively, when compared with a neutral pH value, the use of acidic pH values in the donor compartment increases the P_{app} of a weak acids and decreases the P_{app} of weak bases [12].<br><br>The use of different pH values in the apical and basolateral compartment (usually 6.0/7.4) leads to the establishment of a pH gradient and may have several effects:<br>▪ The pH gradient may activate or inhibit membrane transporters, leading to an increase or decrease in P_{app}. An example is Pep T1 which depends on the pH gradient to transport their substrates [12]<br>▪ An important aspect to consider when using pH gradients is the distinct fraction of compound in the neutral form at both pH values, leading to distinct P_{app} values in the A→B and B→A directions even for compounds that permeate passively. The comparison of these two values of P_{app} cannot therefore be used to evaluate for the presence of active transport [18]
**Concentration effects**	These effects depend strongly on the compound properties and mechanism of transport:<br>▪ When permeation is mediated by a transporter, an increase in compound concentration may cause transporter saturation, and a decrease in transport is expected. The effect on P_{app} depends on whether the transport is in the direction being evaluated of in the opposite direction. That is, when permeation in the A→B direction is being accessed, saturation of an influx transporter leads to a decrease in the net-transport, decreasing P_{app}. In case of saturation of an efflux transporter, the net-transport in the A→B direction increases, increasing the measured P_{app}. The opposite situation occurs if transport in the B→A direction is being accessed [19,20]<br>▪ For compounds that permeate by passive diffusion, the effects depend on the local concentration of the compound in the lipid bilayer. For very lipophilic compounds, a high local concentration may be achieved even for a total concentration in the low μM range. Depending on the compound and membrane, the changes in the physico-chemical properties of the membrane may lead to an increase or decrease in P_{app}. The most common effect for neutral compounds is an increase in membrane fluidity leading to an increase in P_{app}; while charged compounds will change the surface potential of the membrane, leading to a decrease in the compound affinity for the membrane decreasing P_{app}.<br>▪ Very high concentrations can also lead to cell toxicity and/or perturbations of the monolayer integrity, leading to an increase in paracellular P_{app} [21]
**Stirring rate**	Very low stirring rates lead to poor homogenisation of the transport media and result in very thick UWL at the cell monolayer surface. This effect decreases the P_{app}, being most significant for fast permeating compounds [22,23]<br><br>Very high stirring rates lower the UWL but may compromise the cell monolayer integrity, resulting in an increase in the P_{app} of compounds that permeate paracellularly

References

1. Walter, E.; Kissel, T. Heterogeneity in the human intestinal cell line Caco-2 leads to differences in transepithelial transport. *European Journal of Pharmaceutical Sciences* **1995**, *3*, 215-230, doi:10.1016/0928-0987(95)00010-B.
2. Behrens, I.; Kamm, W.; Dantzig, A.H.; Kissel, T. Variation of peptide transporter (PepT1 expression in Caco-2 cells as a function and HPT1) of cell origin. *Journal of Pharmaceutical Sciences* **2004**, *93*, 1743-1754, doi:10.1002/jps.20062.
3. D'Souza, V.M.; Shertzer, H.G.; Menon, A.G.; Pauletti, G.M. High glucose concentration in isotonic media alters Caco-2 cell permeability. *Aaps Pharmsci* **2003**, *5*.
4. Ranaldi, G.; Consalvo, R.; Sambuy, Y.; Scarino, M.L. Permeability characteristics of parental and clonal human intestinal Caco-2 cell lines differentiated in serum-supplemented and serum-free media. *Toxicology in Vitro* **2003**, *17*, 761-767, doi:10.1016/s0887-2333(03)00095-x.
5. DeMarco, V.G.; Li, N.; Thomas, J.; West, C.M.; Neu, J. Glutamine and barrier function in cultured Caco-2 epithelial cell monolayers. *Journal of Nutrition* **2003**, *133*, 2176-2179, doi:10.1093/jn/133.7.2176.
6. Behrens, I.; Kissel, T. Do cell culture conditions influence the carrier-mediated transport of peptides in Caco-2 cell monolayers? *European Journal of Pharmaceutical Sciences* **2003**, *19*, 433-442, doi:10.1016/s0928-0987(03)00146-5.
7. Yu, H.S.; Cook, T.J.; Sinko, P.J. Evidence for diminished functional expression of intestinal transporters in Caco-2 cell monolayers at high passages. *Pharmaceutical Research* **1997**, *14*, 757-762, doi:10.1023/a:1012150405949.
8. Lu, S.; Gough, A.W.; Bobrowski, W.F.; Stewart, B.H. Transport properties are not altered across Caco-2 cells with heightened TEER despite underlying physiological and ultrastructural changes. *Journal of Pharmaceutical Sciences* **1996**, *85*, 270-273, doi:10.1021/js950269u.
9. Hosoya, K.; Kim, K.J.; Lee, V.H.L. Age-dependent expression of P-glycoprotein gp170 in Caco-2 cell monolayers. *Pharmaceutical Research* **1996**, *13*, 885-890, doi:10.1023/a:1016005212640.
10. Markowska, M.; Oberle, R.; Juzwin, S.; Hsu, C.P.; Gryszkiewicz, M.; Streeter, A.J. Optimizing Caco-2 cell monolayers to increase throughput in drug intestinal absorption analysis. *Journal of Pharmacological and Toxicological Methods* **2001**, *46*, 51-55, doi:10.1016/s1056-8719(01)00161-7.
11. Lechanteur, A.; Almeida, A.; Sarmento, B. Elucidation of the impact of cell culture conditions of Caco-2 cell monolayer on barrier integrity and intestinal permeability. *European Journal of Pharmaceutics and Biopharmaceutics* **2017**, *119*, 137-141, doi:10.1016/j.ejpb.2017.06.013.
12. Yamashita, S.; Furubayashi, T.; Kataoka, M.; Sakane, T.; Sezaki, H.; Tokuda, H. Optimized conditions for prediction of intestinal drug permeability using Caco-2 cells. *European Journal of Pharmaceutical Sciences* **2000**, *10*, 195-204, doi:10.1016/s0928-0987(00)00076-2.
13. Saha, P.; Kou, J.H. Effect of solubilizing excipients on permeation of poorly water-soluble compounds across Caco-2 cell monolayers. *European Journal of Pharmaceutics and Biopharmaceutics* **2000**, *50*, 403-411, doi:10.1016/s0939-6411(00)00113-2.
14. Neuhoff, S.; Artursson, P.; Zamora, I.; Ungell, A.L. Impact of extracellular protein binding on passive and active drug transport across Caco-2 cells. *Pharmaceutical Research* **2006**, *23*, 350-359, doi:10.1007/s11095-005-9304-3.
15. Krishna, G.; Chen, K.-j.; Lin, C.-c.; Nomeir, A.A. Permeability of lipophilic compounds in drug discovery using in-vitro human absorption model, Caco-2. *International journal of pharmaceutics* **2001**, *222*, 77-89.

16. Aungst, B.J.; Nguyen, N.H.; Bulgarelli, J.P.; Oates-Lenz, K. The influence of donor and reservoir additives on Caco-2 permeability and secretory transport of HIV protease inhibitors and other lipophilic compounds. *Pharmaceutical Research* **2000**, *17*, 1175-1180, doi:10.1023/a:1026402410783.
17. Yu, H.L.; Huang, Q.R. Investigation of the Absorption Mechanism of Solubilized Curcumin Using Caco-2 Cell Monolayers. *Journal of Agricultural and Food Chemistry* **2011**, *59*, 9120-9126, doi:10.1021/jf201451m.
18. Neuhoff, S.; Ungell, A.-L.; Zamora, I.; Artursson, P. pH-Dependent Bidirectional Transport of Weakly Basic Drugs Across Caco-2 Monolayers: Implications for Drug–Drug Interactions. *Pharmaceutical Research* **2003**, *20*, 1141-1148, doi:10.1023/a:1025032511040.
19. Broeders, J.J.W.; van Eijkeren, J.C.H.; Blaauboer, B.J.; Hermens, J.L.M. Transport of Chlorpromazine in the Caco-2 Cell Permeability Assay: A Kinetic Study. *Chemical Research in Toxicology* **2012**, *25*, 1442-1451, doi:10.1021/tx300221k.
20. Riede, J.; Umehara, K.-I.; Schweigler, P.; Huth, F.; Schiller, H.; Camenisch, G.; Poller, B. Examining P-gp efflux kinetics guided by the BDDCS - Rational selection of in vitro assay designs and mathematical models. *European Journal of Pharmaceutical Sciences* **2019**, *132*, 132-141, doi:10.1016/j.ejps.2019.03.005.
21. Konsoula, R.; Barile, F.A. Correlation of in vitro cytotoxicity with paracellular permeability in Caco-2 cells. *Toxicology in Vitro* **2005**, *19*, 675-684, doi:10.1016/j.tiv.2005.03.006.
22. Karlsson, J.; Artursson, P. A method for the determination of cellular permeability coefficients and aqueous boundary layer thickness in monolayers of intestinal epithelial (Caco-2) cells grown in permeable filter chambers. *International journal of pharmaceutics* **1991**, *71*, 55-64.
23. Korjamo, T.; Heikkinen, A.T.; Waltari, P.; Mönkkönen, J. The Asymmetry of the Unstirred Water Layer in Permeability Experiments. *Pharmaceutical Research* **2008**, *25*, 1714, doi:10.1007/s11095-008-9573-8.
